# Contribution of amyloid deposition from oligodendrocytes in a mouse model of Alzheimer’s disease

**DOI:** 10.1186/s13024-024-00759-z

**Published:** 2024-11-16

**Authors:** Akihiro Ishii, Joseph A. Pathoulas, Omar MoustafaFathy Omar, Yingying Ge, Annie Y. Yao, Tressa Pantalena, Neeraj Singh, John Zhou, Wanxia He, Patrick Murphy, Riqiang Yan, Xiangyou Hu

**Affiliations:** 1 Department of Neuroscience, University of Connecticut Health Center, 263 Farmington Avenue, Farmington, CT 06030-3401 USA; 2 Department of Cell Biology and Vascular Biology Center, University of Connecticut Health Center, 263 Farmington Avenue, Farmington, CT 06030-3401 USA; 3 Department of Neuroscience, University of Connecticut Health Center, 263 Farmington Avenue, Farmington, CT 06030-3401 USA

**Keywords:** BACE1, Amyloid plaques, Oligodendrocytes, ADAM10, IL33, Olig2, ApoE, Myelination

## Abstract

**Background:**

The accumulation of β-amyloid (Aβ) peptides into insoluble plaques is an early pathological feature of Alzheimer’s disease (AD). BACE1 is the sole β-secretase for Aβ generation, making it an attractive therapeutic target for AD therapy. While BACE1 inhibitors have been shown to reduce Aβ levels in people with AD, clinical trials targeting BACE1 have failed due to unwanted synaptic deficits. Understanding the physiological role of BACE1 in individual cell types is essential for developing effective BACE inhibitors for the treatment of AD. Recent single-cell RNA transcriptomic assays revealed that oligodendrocytes are enriched with genes required for generating Aβ. However, the contribution of oligodendrocytes to amyloid plaque burden in AD and the side effects of oligodendrocyte-specific *Bace1* deletion remain to be explored.

**Methods:**

We generated an oligodendrocyte-specific *Bace1* knockout model (*Bace1*^*fl/fl*^;*Olig2-Cre*) to monitor potential disruptions in myelination using standard electron microscopy. Long-term potentiation (LTP) was monitored to measure synaptic integrity. We crossed the *Bace1*^*fl/fl*^;*Olig2-Cre* model with heterozygous *App*^*NL−G−F/wt*^ knock-in AD mice to generate AD mice lacking oligodendrocyte *Bace1* (*Bace1*^*fl/fl*^;*Olig2-Cre; App*^*NL−G−F/wt*^) and examined amyloid plaque number and insoluble Aβ levels and gliosis in these animals. Single nuclei RNA sequencing experiments were conducted to examine molecular changes in response to Bace1 deficiency in oligodendrocytes in the wild type or APP knock-in background.

**Results:**

*Bace1* deletion in oligodendrocytes caused no change in myelin thickness in the corpus callosum but a marginal reduction in myelin sheath thickness of the optic nerve. Synaptic strength measured by LTP was not different between *Bace1*^*fl/fl*^;*Olig2-Cre* and age-matched *Bace1*^*fl/fl*^ control animals, suggesting no major effect on synaptic plasticity. Intriguingly, deletion of *Bace1* in 12-month-old heterozygous AD knock-in mice (*Bace1*^*fl/fl*^;*Olig2-Cre; App*^*NL−G−F/wt*^ mice) caused a significant reduction of amyloid plaques by ~ 33% in the hippocampus and ~ 29% in the cortex compared to age-matched AD mice (*Bace1*^*fl/fl*^;*App*^*NL−G−F/wt*^). Insoluble Aβ_1–40_ and Aβ_1–42_ levels were reduced comparably while more astrocytes and microglia were observed in surrounding amyloid plaques. Unbiased single-nuclei RNA sequencing results revealed that deletion of oligodendrocyte *Bace1* in *APP*^*NL−G−F/wt*^ knock-in mice increased expression of genes associated with Aβ generation and clearance such as *ADAM10*, *Ano4*,* ApoE*, *Il33*, and *Sort1*.

**Conclusion:**

Our results provide compelling evidence that the amyloidogenic pathway in oligodendrocytes contributes to Aβ plaque formation in the AD brain. While specifically targeting BACE1 inhibition in oligodendrocytes for reducing Aβ pathology in AD is likely challenging, this is a potentially explorable strategy in future studies.

**Supplementary Information:**

The online version contains supplementary material available at 10.1186/s13024-024-00759-z.

## Introduction

Alzheimer’s disease (AD) is the most common neurodegenerative disease and manifests clinically as a gradual decline in memory and cognitive function [61]. The abnormal accumulation of neurotoxic β-amyloid peptides (Aβ) is a critical first step in AD pathogenesis, leading to hyperphosphorylated tau protein formation, neuroinflammation, and neuronal death [27, 64]. Aβ is generated from the enzymatic processing of the transmembrane amyloid precursor protein (APP). Full-length APP (APP-fl) is first cleaved by either α-secretase, which generates an 83 amino acid C-terminal fragment (CTF-83), or β-site APP-cleaving enzyme 1 (BACE1) which generates a 99 amino acid C-terminal fragment (CTF-99) [26, 53, 56, 63]. The enzyme γ-secretase subsequently cleaves CTF-99, releasing Aβ species, including Aβ_1–42_, which can more readily oligomerize and deposit as insoluble plaques (Li et al., 2000; Wolfe et al., 1999).

Given its role in Aβ production, BACE1 is an attractive pharmacological target for AD therapies [19, 35]. BACE1 inhibitors have been shown to effectively lower Aβ levels in animal AD models [22] and human clinical trials [13, 39]. Unfortunately, mice with global or neuron-specific *Bace1* knockout exhibit defects in neurotransmitter release, synaptic plasticity, and neurogenesis [5, 8, 32, 40, 57, 68]. Moreover, despite promising reduction in Aβ pathology in preclinical and early clinical studies, BACE1 inhibitors failed to improve cognition in phase II/III clinical trials [2, 10]. This highlights the importance of neuronal BACE1 in maintaining normal synaptic functioning and may explain the lack of cognitive benefit seen with BACE1 inhibitor treatment. Therefore, there is an urgent need to identify alternative BACE1 targeting strategies.

Recently, studies have shown that BACE1 expression in glial cells can alter Aβ pathology in AD mouse models [21, 45, 51, 67]. Notably, single-cell RNA sequencing analysis indicates that *Bace1* is highly expressed in oligodendrocytes but at slightly lower levels than neurons [51]. Oligodendrocytes are responsible for myelinating axons in the central nervous system (CNS) and maintaining proper neuronal transmission [3, 36]. Interestingly, oligodendrocyte dysfunction and demyelination have been reported to occur early in AD pathogenesis and correlate with disease severity [11, 12, 38, 55]. We asked whether BACE1 in oligodendrocytes would be an important target for AD therapy.

In this study, we investigated the function of BACE1 in oligodendrocytes and how *Bace1* deletion in oligodendrocytes affects AD amyloid pathology. First, we generated a novel oligodendrocyte-specific *Bace1* knockout model (*Bace1*^*fl/fl*^;*Olig2-Cre*) by utilizing the oligodendrocyte transcription factor 2 (Olig2) Cre driver line [65]. Considering the known role of BACE1 in myelination [24, 60] and synaptic functions [57], we analyzed myelination in the central nervous system (CNS) and measured hippocampal long-term potentiation (LTP). We observed slight hypomyelination in the optic nerve which, was not present in the corpus callosum. No difference was noted in Schaffer collateral LTP in *Bace1*^*fl/fl*^;*Olig2-Cre* mice compared to age-matched controls. Next, to determine the contribution of oligodendrocyte BACE1 to amyloid plaque formation, we generated *Bace1*^*fl/fl*^;*Olig2-Cre; App*^*NL−G−F/wt*^ mice by breeding *Bace1*^*fl/fl*^;*Olig2-Cre* mice with heterozygous APP knock-in (*Bace1*^*fl/fl*^;*App*^*NL−G−F/wt*^*)* AD mice. Unexpectedly, we detected significantly reduced levels of Aβ plaques and insoluble Aβ levels in 12-month-old *Bace1*^*fl/fl*^;*Olig2-Cre; App*^*NL−G−F/wt*^ mice compared to *Bace1*^*fl/fl*^;*App*^*NL−G−F/wt*^ mice. This result is in line with two recent publications [42, 48], supporting the same conclusion that oligodendrocytes participate in amyloid deposition. Further, single-nuclei RNA sequencing (snRNA-Seq) analysis revealed increased expression of *ADAM10*, *Ano4*,* ApoE*, *Il33*, and *Sort1* when *Bace1 was* deleted in oligodendrocytes of *APP*^*NL−G−F/wt*^ knock-in mice; elevation of these genes is related to either precluding Aβ generation or facilitating Aβ clearance. Hence, we demonstrated an important contribution of oligodendrocytes to amyloid pathology in AD mouse brains.

## Materials and methods

### Animals

We crossed *Bace1* conditional knockout (*Bace1*^*fl/fl*^) mice [22] with *Olig2-Cre* mice (JAX:025567) [65] to obtain *Bace1*^*fl/fl*^;*Olig2-Cre* mice. We also crossed *Bace1*^*fl/fl*^ mice with heterozygous *App*^*NL−G−F/wt*^ knock-in AD mice (RIKEN Center for Brain Science, Japan) to generate *Bace1*^*fl/fl*^;*App*^*NL−G−F/wt*^ mice. Finally, we crossed *Bace1*^*fl/fl*^;*Olig2-Cre* with *Bace1*^*fl/fl*^; *App*^*NL−G−F/wt*^ mice to obtain *Bace1*^*fl/fl*^;*Olig2-Cre; App*^*NL−G−F/wt*^ mice. All mice were maintained on a C57/Bl6J background and housed on a 12-hour light/12-hour dark cycle with access to food and water *ad libitum* and both sexes were used. All animal use and procedures were performed according to the Institutional Animal Care and Use protocols at UConn Health, Farmington, and in compliance with the guidelines established by the Guide for the Care and Use of Laboratory Animals, as adopted by the National Institutes of Health.

### Immunohistochemistry

Mice brains were surgically removed and cut mid-sagittally into equal halves. One half of the brain was fixed in 4% paraformaldehyde for 24 h and then immersed in 20% sucrose overnight at 4 °C and then embedded with optimal cutting temperature compound (OCT). The other half was used for western and ELISA analysis. Brains were sectioned sagittal into 16 μm-thick sections on a cryostat microtome (Thermo HM525 NX). Sections on slides were washed in PBS 3× for 5 min to remove OCT and then permeabilized with 0.03% H_2_O_2_/0.3% Triton X-100 in PBS for 30 min, followed by washing with PBS (3× for 5 min). Antigen retrieval was performed by microwaving the sections in 0.05 M citrate-buffered saline (pH 6.0) for 3 min. The sections were blocked with 5% normal goat serum and incubated with the primary antibody 6E10 (1:1000 dilution, AB_2564652, BioLegend). After washing with PBS (3× for 5 min), the sections were incubated with universal biotinylated anti-mouse/rabbit IgG (1:200, Vector Laboratories) at room temperature for 2 h. Washing with PBS (3× for 5 min), the sections were incubated with avidin-biotin peroxidase complex (1:200, Vector Laboratories) at room temperature for 1 h and developed with 0.05% DAB (Sigma) with 0.01% H_2_O_2_ in PBS for 5 min. Then, the slides were mounted on a coverslip with 60% glycerol followed by standard immunohistochemical staining procedures. Antibodies for amyloid plaques and glial cells are marked by 6E10 (1:1000, Covance Research Products Inc Cat# SIG-39330-200, RRID: AB_662804), IBA1 (1:1000, AB_839504, Fuji Wako Chemical USA), GFAP (1:1000, AB_2631098, Cell Signaling). Dystrophic neurites are marked by RTN3 R458 antibody [25].

### Isolation *O4*^*+*^*immature and mature oligodendrocytes*

O4^+^ immature and mature oligodendrocytes were isolated from the forebrain in Postnatal day 13 (P13) pups (5 pups per group in *Bace1*^*fl/fl*^, *Bace1*^*fl/fl*^;*Olig2-Cre*, *Bace1*^*fl/fl*^; *App*^*NL−G−F/wt*^, and *Bace1*^*fl/fl*^;*Olig2-Cre; App*^*NL−G−F/wt*^) using anti-O4 microbeads (MiltenyiBiotec, catalog no. 130-094-543) and adult brain dissociation kits (MiltenyiBiotec, catalog no. 130-107-677). Briefly, forebrains were dissociated into a single-cell suspension; myelin, cell debris, and erythrocytes were removed subsequently; and cells were immunolabeled with anti-O4 microbeads. The cell suspension was allowed to pass though the magnetic column and retained O4^+^ cells from the column, which were flushed out and washed with PBS for western blotting.

### Western blotting

O4^+^ cells and brain tissues were homogenized in radioimmunoprecipitation assay (RIPA) buffer (50 mM Tris–HCl, pH 7.4, 1% NP-40, 0.25% sodium deoxycholate, 150 mM NaCl, 1 mM EDTA, 1 mM NaF, 1 mM Na_3_VO_4_, and a protease inhibitor cocktail [Roche]) and centrifuged at 13,200 rpm for 90 min. Protein concentrations were determined using a bicinchoninic acid (BCA) assay kit. Equal amounts of protein were loaded and resolved on 4 to 12% SDS-polyacrylamide gels (NuPAGE system, Life Technologies). Subsequently, blots were transferred to nitrocellulose membranes at 100 V for 2 h. The membranes were blocked with 5% BSA for 1 h at RT. The membranes were probed with the following primary antibodies at the noted dilutions: 1:1,000 APP-C (AB_258409, Sigma); 1:1,000 BACE1(gift from Huaibin Cai, NIH); 1:500 Olig2 (AB_2157541, Proteintech); 1:1,000 MBP (AB_2799920, Cell Signaling); 1:5,000 PLP (gift from Bruce Trapp, Cleveland Clinic);1:1000 NeuN (AB_2298772, Millipore); 1:50,000 actin (AB_476744, Sigma); 1:5,00 ADAM10 (AB_2242320, Millipore) Aβ42 (1:1000, AB_2798671, Cell Signaling). After 24 h primary incubation at 4^o^C, blots were washed extensively, incubated with HRP-conjugated secondary antibodies, and visualized using enhanced chemiluminescence (Thermo Scientific). The antibody-bound protein blots were detected by an iBright 1500 imaging system (Invitrogen). For quantification purposes, band intensities of immunoblots were analyzed using ImageJ software (National Institutes of Health). Original blot images can be found in the supplemental file.

### Fluorescent in situ hybridization

In situ hybridization was performed using the RNAscope Multiplex Fluorescent v2 kit (ACD Bio, Cat. No. 323100) and the kit-described procedures. Briefly, fixed-frozen brains were sectioned sagittal into 16 μm-thick sections on a cryostat microtome (Thermo HM525 NX). The sections were post-fixed in 4% paraformaldehyde and dehydrated in 50%, 70%, and 100% ethanol. After treatment with hydrogen peroxide and the target retrieval, sections were digested with Protease III for 30 min at 40 °C. After washing with wash buffer, sections were hybridized for 2 h at 40 °C with the following probes: *mBace1*-C2, *mSyp*-C3 (neuronal marker), and *mMbp*-C3 (oligodendrocyte marker). The probes were then amplified by sequentially incubating with the kit reagents AMP1, AMP2, and AMP3. Finally, the sections were developed by sequentially incubating with an appropriate HRP linked to the specific probe channel, fluorophore dye, and HRP blocker. Images were captured using Zeiss LSM800 confocal microscopy.

### Quantification of amyloid plaque load

Serial sagittal brain sections starting from the beginning of the hippocampus were selected at 10-section intervals. Sections were probed with Aβ monoclonal antibody 6E10, which recognizes the first 16 residues of Aβ, and stained with DAB as described above. Images were captured with a Keyence fluorescence microscope (Keyence, BZ-X810). Plaque counting in the cortex and hippocampus was conducted using ImageJ software (National Institutes of Health).

### Quantification of Aβ_**1−42**_ and Aβ_1−40_ by ELISA

Insoluble Aβ_1–42_ was prepared from frozen hippocampal tissues by the guanidine hydrochloride method [22]. Levels of insoluble Aβ_1–42_ and Aβ_1–40_ were quantified by the human Aβ_42_ ultrasensitive ELISA kit (ThermoFisher, cat # KHB3544) and human Aβ_40_ ELISA kit (ThermoFisher, cat # KHB3481) according to kit instructions. Results were obtained from 12-month-old male (6 *Bace1*^*fl/fl*^;*App*^*NL−G−F/wt*^ and 8 *Bace1*^*fl/fl*^;*Olig2-Cre; App*^*NL−G−F/wt*^) and female (3 *Bace1*^*fl/fl*^;*App*^*NL−G−F/wt*^ and 6 *Bace1*^*fl/fl*^;*Olig2-Cre; App*^*NL−G−F/wt*^) mice. Numbers and size of GFAP^+^-reactive astrocytes and IBA1^+^-microglia in surrounding amyloid plaques were chosen for quantification by utilizing ImageJ software (NIH).

### Quantification of g-ratios

Myelin sheath thickness was examined according to the procedure described in our previous study [24]. The myelinated axon circumference was measured by digitally tracing the inner and outer layers of the myelinated fiber using Photoshop CS6 with measurement tools. (Adobe). The g-ratio was calculated as the inner-to-outer diameter of a myelinated axon.

### LTP recordings

LTP recordings on hippocampal slices were performed according to previously described procedures using a MED-A64HE1S head amplifier and a MED-A64MD1 main amplifier, run by Mobius software [9, 22]. Upon obtaining horizontal hippocampal slices from three female 12-month-old *Bace1*^*fl/fl*^ and *Bace1*^*fl/fl*^;*Olig2-Cre* mice, the prepared slices were then placed onto the center of an MED probe (MED-P515A; AutoMate Scientific) with continuous perfusion of artificial cerebrospinal fluid consisting of (in mM): 115 NaCl, 2 KCl, 1.25 KH2PO4, 1.0 MgSO4, 2.0 CaCl2, 26 NaHCO3, 10 glucose, and 1.0 L-Ascorbic acid and bubbling of 95% O_2_/5% CO_2_. The device has an array arranged in an 8 × 8 pattern of 64 planar microelectrodes across a hippocampal slice. Each electrode used for data acquisition and analysis was 20 μm × 20 μm with an interelectrode distance of 150 μm. Schaffer collateral-to-CA1 synapses were analyzed for LTP assays. Field excitatory postsynaptic potentials (fEPSPs) caused by theta burst stimulation were recorded at a 20-kHz sampling rate within the CA1 subregion of the hippocampus. Control fEPSPs were recorded for at least 10 min before the conditioning stimulation using a response ~ 50% of the maximum. After a stable baseline was established, LTP was induced with three trains of 100 Hz for 1 s with an intertrain interval of 20 s. Field potential amplitudes were then measured. Synaptic strength was evaluated by measuring changes in the fEPSP amplitude relative to baseline.

### Single nuclear transcript generation

Hippocampi and cortices were extracted from 8 female mice at the age of ~ 5 months, with 2 mice each in *Bace1*^*fl/fl*^, *Bace1*^*fl/fl*^;*Olig2-Cre*,* Bace1*^*fl/fl*^;*App*^*NL−G−F/wt*^, and *Bace1*^*fl/fl*^;*Olig2-Cre; App*^*NL−G−F/wt*^, and were snap-frozen on dry ice. Tissues were then homogenized and lysed in buffer containing 0.01% tween/0.01% NP-40 on ice. A debris removal (Miltenyi Biotec, Germany) step was included to remove myelin debris, and nuclei were washed and suspended in PBS containing 0.04% BSA and RNase inhibitor (Invitrogen) and immediately processed as follows. Nuclei count and viability were assessed on a LUNA FX7 automated cell counter (Logos Biosystems), and up to 32,000 nuclei from each suspension were loaded onto one lane of a 10x Genomic Chip M. Single cell capture, barcoding and library preparation were performed using the 10x Genomics Chromium X platform [66] version 3.1 NEXTGEM chemistry and according to the manufacturer’s protocol (#CG000416). cDNA and libraries were checked for quality by Tapestation 4200 (Agilent) and Qubit Fluorometer (ThermoFisher), quantified by KAPA qPCR, and sequenced on an Illumina NovaSeq X Plus 100 cycle 10B flow cell, with a 28-10-10-90 asymmetric read configuration, targeting 12,000 barcoded nuclei with an average sequencing depth of 70,000 read pairs per nucleus. Illumina base call files for all libraries were converted to FASTQs using bcl2fastq v2.20.0.422 (Illumina) and FASTQ files associated with the gene expression libraries were aligned to the GRCm38 reference assembly with vM23 annotations from GENCODE (10x Genomics mm10 reference 2020-A) using the version 7.2.0 CellRanger count pipeline (10x Genomics), producing a digital cell by gene counts matrix corresponding to each input suspension.

### Dimensionality reduction and clustering

Data were then analyzed in an R (v4.3.0) environment using Seurat v4.4.0. We created Seurat objects for each library after removing nuclei with less than 200 features and features occurring in fewer than three nuclei. The raw counts matrix was filtered using cutoff values of mitochondrial transcripts below 5% and between 100 and 7500 unique features. The expression profiles of each cell using the 2000 most variable genes as measured by dispersion [49, 66] were used for neighborhood graph generation and dimensionality reduction with UMAP [4, 58]. Clustering with Louvain algorithm, cell type annotation, and differential expression was performed ad hoc on a per-cluster basis using the Seurat v4.0 R toolkit [20]. Cell types in each cluster were assigned with the marker genes, excitatory neurons (*Slc17a7*), inhibitory neurons (*Gad2*), astrocytes (*Aqp4*,* Clu*), microglia (*Cx3cr1*,* Hexb*), endothelial (*Cldn5*,* Vtn*), macrophage (*Marc1*), pericytes (*Atp13a5*), ependymal (*Kl*), OPC (*Pdgfra*) and OLs (*Mog*) (Supplemental Figure S1).

Downstream analysis of single-nuclei RNA sequencing data was performed using Scanpy (v1.9.3). Filtered data output from cellranger from eight samples were read into Scanpy, annotated, and concatenated into a single AnnData object. Quality control involved filtering out cells with fewer than 50 genes and mitochondrial content above 5%. Genes expressed in fewer than 5 cells were also removed, followed by normalization and log transformation. Doublets were identified using Scrublet with a batch-specific threshold of 0.15 and removed. Highly variable genes were selected with specific cutoffs for mean and dispersion. Principal component analysis (PCA) was performed using these genes, and batch effects were corrected using Harmony [29]. Dimensionality reduction was achieved with UMAP, and clustering was performed using the Leiden algorithm at a resolution of 0.8. Differential gene expression tables were generated using rank_genes_groups function in scanpy using Wilcoxon test.

### Gene ontology (GO) analysis

Gene ontology term enrichment was performed using the R package clusterProfiler 4.6.2 [62]. The mapIds() function in the org.Mm.eg.db package was used to convert gene symbols to Ensmbl IDs. The functions enrichGO from clusterProfiler were then used to enrich for gene ontology terms from GO databases (biological process). A p-value cutoff of 0.05 was used to determine significant GO terms.

### Statistical analysis

All results are expressed as means ± standard deviation (SD). Statistical analyses were performed using GraphPad Prism 6.0 software (GraphPad Software, San Diego). Welch’s *t* tests were used in the case of a significant F-test. Two-tailed, unpaired Student’s *t* tests were used for all other comparisons. Differences with *p < 0.05, **p < 0.01, and ***p < 0.001 were considered significant.

## Results

### Targeted *Bace1* deletion in mouse oligodendrocytes

**Fig. 1:**
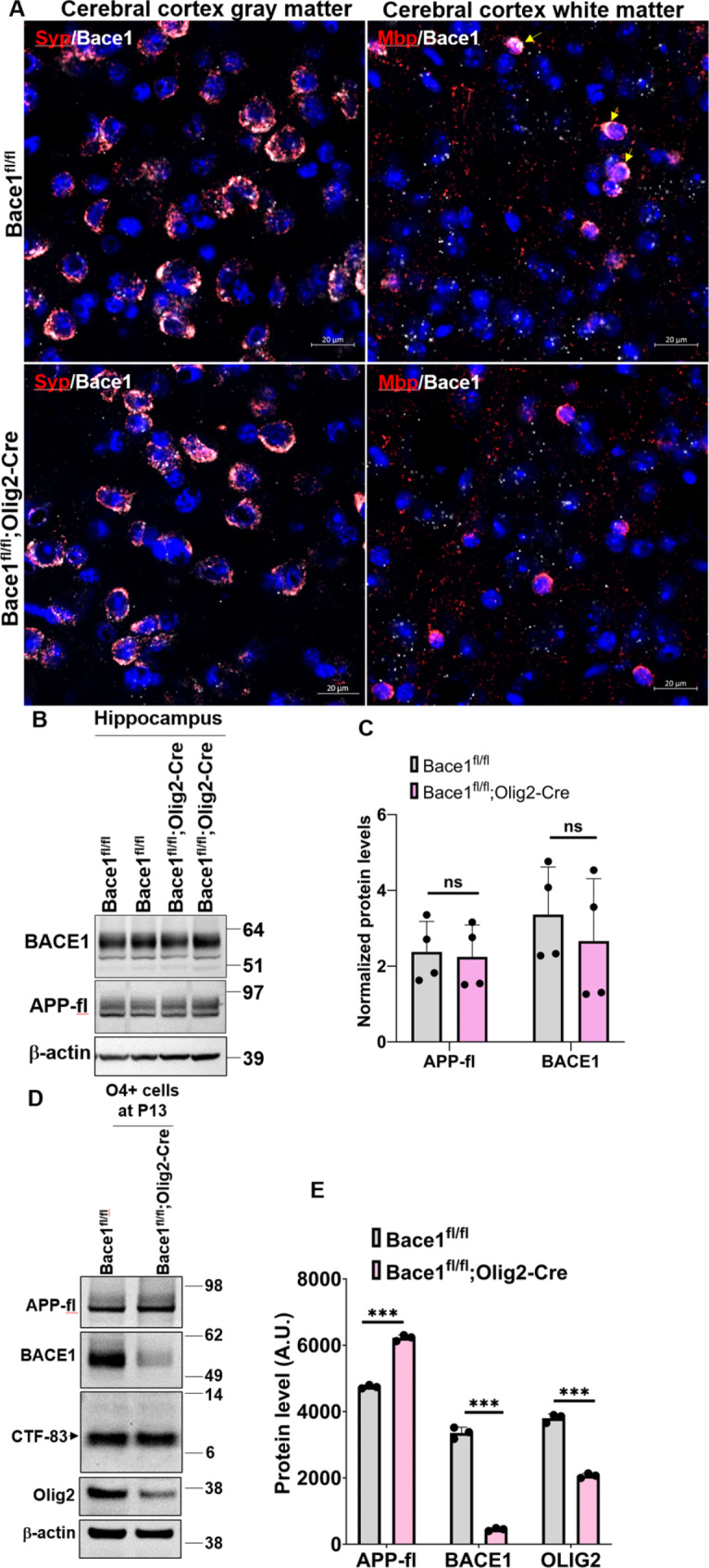
**BACE1 was abolished in*Bace1***^***fl/fl***^;***Olig2-Cre*mice.** (**A**) Representative images of fluorescent in situ hybridization to double-probing *Bace1mRNA* (white) / *Syp mRNA* (red, neuronal marker) and *Bace1* mRNA (white) / *Mbp* mRNA (red, oligodendrocyte marker) in the gray matter and white matter of cerebral cortex in 4-month-old *Bace1*^*fl/fl*^ and *Bace1*^*fl/fl*^;*Olig2-Cre* brains (*N* = 3). Scale bar, 20 μm. (**B**) Immunoblot analysis of BACE1 and full-length APP in 4-month-old *Bace1*^*fl/fl*^ and *Bace1*^*fl/fl*^;*Olig2-Cre* hippocampi. Antibody to β-actin was used as loading control. Blot measurements are in kilodaltons (kDa). (**C**) Bar graph shows quantification of relative protein levels based on the blot shown in B. *N* = 3 independent experiments. (**D**) Immunoblot analysis of BACE1, full-length APP, CTF-83, and Olig2 as measured in the O4^+^ cells which were isolated from P13 forebrains in *Bace1*^*fl/fl*^ and *Bace1*^*fl/fl*^;*Olig2-Cre* pups. Antibody to β-actin was used as loading control. Blot measurements are in kilodaltons (kDa). (**E**) Bar graph shows quantification of relative protein levels based on the blot shown in D. *N* = 3 independent experiments; five pups were used to isolate O4^+^ immature and mature oligodendrocytes in each group. ***P < 0.001, two-tailed Student’s *t* test. Values are expressed as mean ± SD

Global and neuron-specific *Bace1* deletion models exhibit significant synaptic side effects and deficits in synaptogenesis and maturation of oligodendrocytes [5]. However, the impact of *Bace1* deletion specifically in oligodendrocytes and how it impacts their cellular function is unknown. To evaluate this, we crossed *Olig2-Cre* mice [65], JAX:025567) with *Bace1* conditional knockout (*Bace1*^*fl/fl*^) mice [22] to obtain *Bace1*^*fl/fl*^;*Olig2-Cre* mice. To evaluate deletion of *Bace1* in oligodendrocytes, we performed RNA in situ hybridization fluorescent assays to examine *Bace1* expression in *Bace1*^*fl/fl*^ control and *Bace1*^*fl/fl*^;*Olig2-Cre* mouse brains. As shown, *Bace1* was highly expressed in both *Syn*^*+*^ neurons and *Mbp*^*+*^ oligodendrocytes in the cerebral cortex of 4-month-old *Bace1*^*fl/fl*^ control mice (Fig. 1A). In *Bace1*^*fl/fl*^;*Olig2-Cre* brains, *Bace1* was barely detectable in oligodendrocytes, while neuronal* Bace1* in both *Bace1*^*fl/fl*^;*Olig2-Cre* and *Bace1*^*fl/fl*^ mice was comparable (Fig. 1A).

Since neuronal BACE1 was not obviously affected, BACE1 protein levels in *Bace1*^*fl/fl*^;*Olig2-Cre* hippocampal tissues, which contained BACE1 from all hippocampal cells, were not discernibly altered compared to that in *Bace1*^fl/fl^ controls (Fig. 1B-C). Consistently, full-length APP (APP-fl) levels were not altered. For a more specific comparison of proteins levels in oligodendrocytes, we isolated O4^+^ immature and mature oligodendrocytes from postnatal day 13 (P13) mouse forebrains. BACE1 was significantly reduced while APP-fl was visibly increased in *Bace1*^*fl/fl*^;*Olig2-Cre* mice compared to *Bace1*^*fl/fl*^ controls (Fig. 1D-E), indicating abrogated cleavage of APP. Interestingly, α secretase-cleaved APP cleavage product, CTF-83, was not different between the groups (Fig. 1D-E). Since APP in WT mice is predominantly cleaved by α secretase, a small increase in CTF83 was likely not notable. We also found OLIG2 levels were reduced by ~ 50% in *Bace1*^*fl/fl*^;*Olig2-Cre* compared to *Bace1*^*fl/fl*^, likely because of one copy of *Cre* recombinase being inserted into the *Olig2* gene, leading to the reduction of OLIG2 levels [65].

### Deletion of *Bace1* in oligodendrocytes reduces amyloid plaque formation in AD mice

**Fig. 2:**
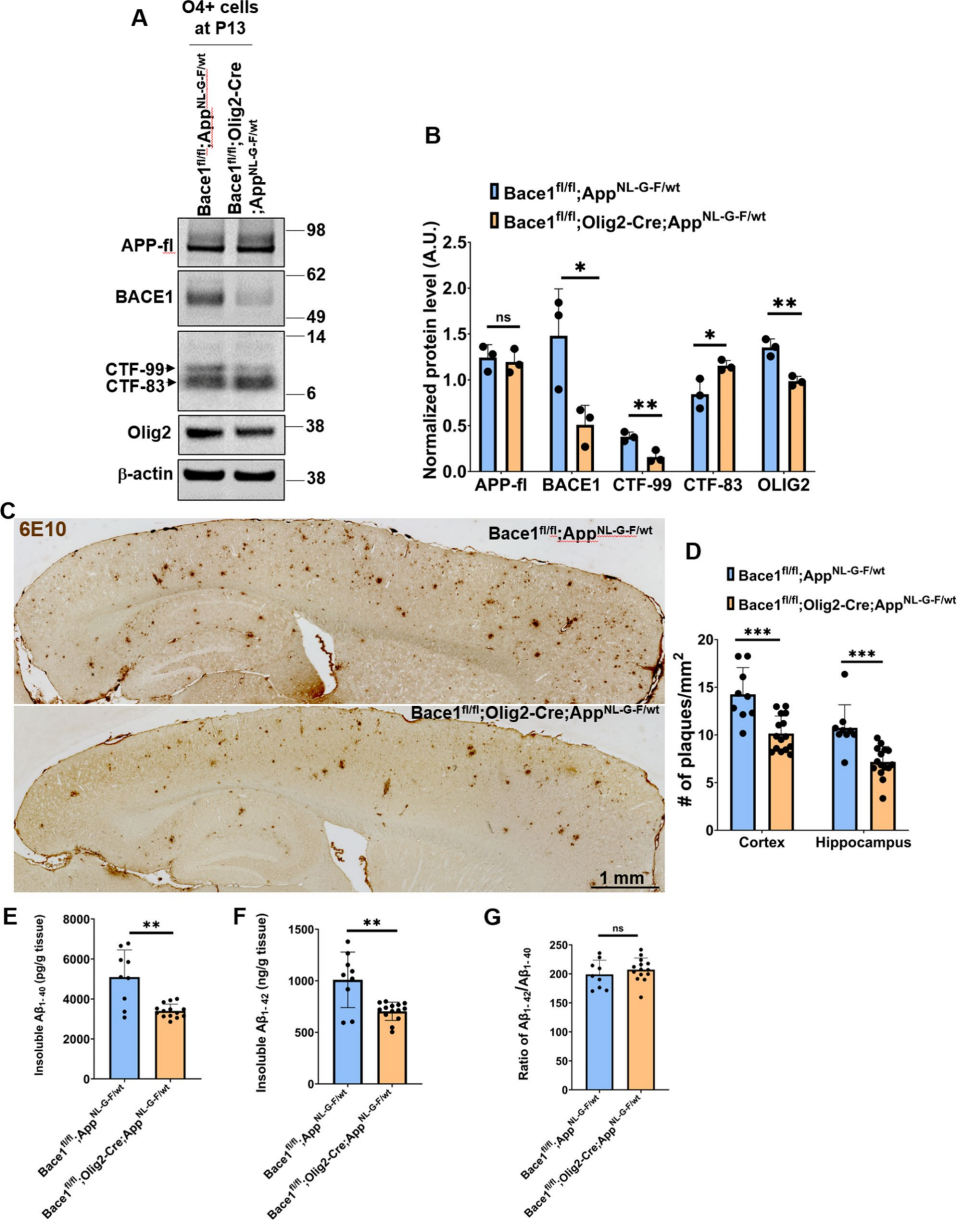
**Plaque load was reduced in 12-month-old*Bace1***^***fl/fl***^;***Olig2-Cre; App***^***NL−G−F/wt***^**brains.** (**A**) Immunoblot analysis of full-length APP, BACE1, CTF-99/83, and Olig2 as measured in the O4^+^ cells which were isolated from P13 forebrains in *Bace1*^*fl/fl*^; *App*^*NL−G−F/wt*^ and *Bace1*^*fl/fl*^;*Olig2-Cre; App*^*NL−G−F/wt*^ pups. Antibody to β-actin was used as loading control. Blot measurements are in kilodaltons (kDa). (**B**) Bar graph shows quantification of relative protein levels based on the blot shown in A. *N* = 3 independent experiments; five pups were used to isolate O4^+^ immature and mature oligodendrocytes in each group. (**C**) Representative images of DAB staining of amyloid plaques using 6E10 monoclonal antibody in 12-month-old *Bace1*^*fl/fl*^; *App*^*NL−G−F/wt*^ and *Bace1*^*fl/fl*^;*Olig2-Cre; App*^*NL−G−F/wt*^. *N* = 5 male and 4 female *Bace1*^*fl/fl*^; *App*^*NL−G−F/wt*^ mice, and 9 male and 6 female *Bace1*^*fl/fl*^;*Olig2-Cre; App*^*NL−G−F/wt*^; five sections were selected in every 10th per mouse (Scale bar, 1 mm). (**D**) Quantification of plaque load in the cerebral cortex and hippocampus. Total insoluble Aβ_1 − 40_ (**E**) and Aβ_1 − 42_ (**F**) and the ratio of Aß_1-42_/Aß_1-40_ (**G**) from the hippocampal tissues in 12-month-old *Bace1*^*fl/fl*^; *App*^*NL−G−F/wt*^ and *Bace1*^*fl/fl*^;*Olig2-Cre; App*^*NL−G−F/wt*^ were extracted and measured by the human Aβ_42_ ultrasensitive ELISA kit, *N* = 6 male and 3 female *Bace1*^*fl/fl*^; *App*^*NL−G−F/wt*^ mice, and 8 male and 6 female *Bace1*^*fl/fl*^;*Olig2-Cre; App*^*NL−G−F/wt*^. ***P < 0.001, **P < 0.01, *P < 0.05, two-tailed Student’s *t* test

Since genes in amyloidogenic pathways are expressed in oligodendrocytes, we asked whether *Bace1* deletion in oligodendrocytes would affect amyloid deposition in AD mouse brains. To this end, we crossed *Bace1*^*fl/fl*^;*Olig2-Cre* mice with *Bace1*^*fl/fl*^;*App*^*NL−G−F/wt*^ mice [47] to generate an AD mouse line lacking oligodendrocyte *Bace1* (*Bace1*^*fl/fl*^;*Olig2-Cre; App*^*NL−G−F/wt*^). O4^+^ immature and mature oligodendrocytes were similarly isolated from P13 mouse forebrains to evaluate APP processing with or without Bace1 deletion. We showed that BACE1 levels were significantly reduced in *Bace1*^*fl/fl*^;*Olig2-Cre; App*^*NL−G−F/wt*^ cells and correlated with decreased the levels of CTF-99, the BACE1-cleaved APP cleavage product, in *Bace1*^*fl/fl*^;*Olig2-Cre; App*^*NL−G−F/wt*^ cells, while CTF83 levels were higher (Fig. 2A-B). OLIG2 levels were reduced by ~ 50% in *Bace1*^*fl/fl*^;*Olig2-Cre; App*^*NL−G−F/wt*^ compared to *Bace1*^*fl/fl*^;*App*^*NL−G−F/wt*^ controls.

Next, we stained amyloid plaques in 12-month-old *Bace1*^*fl/fl*^;*App*^*NL−G−F/wt*^ and *Bace1*^*fl/fl*^;*Olig2-Cre; App*^*NL−G−F/wt*^ mice. We found a visible reduction of plaque deposition in both the cerebral cortex and hippocampus of *Bace1*^*fl/fl*^;*Olig2-Cre; App*^*NL−G−F/wt*^ mice (Fig. 2C). In *Bace1*^*fl/fl*^;*Olig2-Cre; App*^*NL−G−F/wt*^ mice, we noted that the plaque number was reduced by ~ 29% in the cerebral cortex (Figs. 2D and 14.27 ± 2.79 plaques per mm^2^ in *Bace1*^*fl/fl*^;*App*^*NL−G−F/wt*^, *N* = 9, vs. 10.14 ± 1.81 in *Bace1*^*fl/fl*^;*Olig2-Cre; App*^*NL−G−F/wt*^, *N* = 15, *P* < 0.001), and by ~ 33% in the hippocampus (Figs. 2D and 10.74 ± 2.42 in *Bace1*^*fl/fl*^;*App*^*NL−G−F/wt*^ vs. 7.17 ± 1.61 in *Bace1*^*fl/fl*^;*Olig2-Cre; App*^*NL−G−F/wt*^, *P* < 0.001). In line with plaque reduction, both insoluble Aβ_1–40_ (Figs. 2E and 5092.78 ± 1360.73 tissue in *Bace1*^*fl/fl*^;*App*^*NL−G−F/wt*^, *N* = 9 vs. 3563.66 ± 631.04 pg/g tissue in *Bace1*^*fl/fl*^;*Olig2-Cre; App*^*NL−G−F/wt*^, *N* = 14, *P* < 0.01) and Aβ_1–42_ levels were reduced by ~ 30% in hippocampal tissues of 12-month-old *Bace1*^*fl/fl*^;*Olig2-Cre; App*^*NL−G−F/wt*^ mice when compared to age-matched *Bace1*^*fl/fl*^;*App*^*NL−G−F/wt*^ littermates (Figs. 2F and 1009.22 ± 269.19 ng/g tissue in *Bace1*^*fl/fl*^;*App*^*NL−G−F/wt*^, *N* = 9, vs. 704.19 ± 88.66 ng/g tissue in *Bace1*^*fl/fl*^;*Olig2-Cre; App*^*NL−G−F/wt*^, *N* = 14, *P* < 0.01). The ratio of Aβ_1–42_ over Aβ_1–40_ was not significantly altered (Figs. 2G and 199.15 ± 24.33 in *Bace1*^*fl/fl*^;*App*^*NL−G−F/wt*^ vs. 207.23 ± 20.14 in *Bace1*^*fl/fl*^;*Olig2-Cre; App*^*NL−G−F/wt*^; *P* = 0.3962). There was no detectable gender effect on Aβ production or amyloid plaque load in both *Bace1*^*fl/fl*^;*App*^*NL−G−F/wt*^ and *Bace1*^*fl/fl*^;*Olig2-Cre; App*^*NL−G−F/wt*^ mouse brains (Supplemental Fig. 1A-B).

**Fig. 3:**
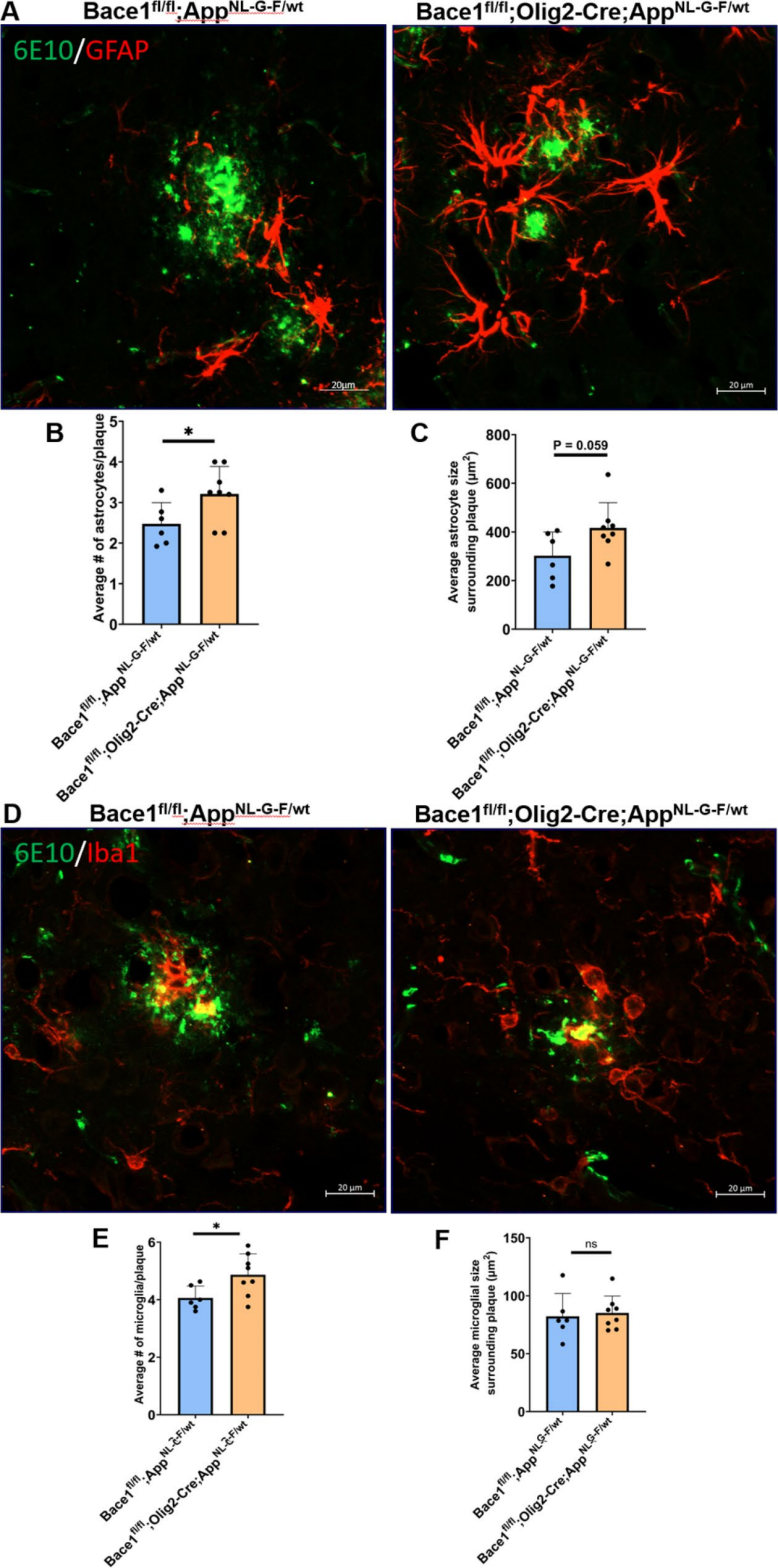
**More astrocytes and microglia in surrounding amyloid plaques in AD mice with Bace1 deletion in oligodendrocytes.** (**A**) Representative images show amyloid plaques (green) marked by 6E10 antibody and astrocytes (red) marked by glial fibrillary acidic protein (GFAP) antibody in the cerebral cortex of 12-month-old *Bace1*^*fl/fl*^;*App*^*NL−G−F/wt*^ and *Bace1*^*fl/fl*^;*Olig2-Cre; App*^*NL−G−F/wt*^ mice. (**B**-**C**) Quantification of number and size of reactive astrocytes surrounding amyloid plaque. (**D**) Representative images of amyloid plaques (green) surrounded by IBA1-labeled microglia (red) in the same age group of mice. (**E**-**F**) Quantification of number and size of activated microglia surrounding amyloid plaque. *N* = 6 mice in *Bace1*^*fl/fl*^;*App*^*NL−G−F/wt*^ and 8 mice in *Bace1*^*fl/fl*^;*Olig2-Cre; App*^*NL−G−F/wt*^ mice (79 plaques in Bace1^fl/fl^;App^NL−G−F/wt^ and 80 in *Bace1*^fl/fl^;Olig2-Cre; App^NL−G−F/wt^ were analyzed. *P < 0. 05, two-tailed Student’s *t* test). Values are expressed as mean ± SD. Scale bar in A and D, 20 μm

One intriguing observation was that a higher number of astrocytes were found to surround neuritic plaques in *Bace1*^*fl/fl*^;*Olig2-Cre; App*^*NL−G−F/wt*^ compared to that in *Bace1*^*fl/fl*^;*App*^*NL−G−F/wt*^ mice (Fig. 3A; quantified in 3B: 3.21 ± 0.67 vs. 2.47 ± 0.52 per plaque, *P < 0.05). The overall astrocyte size near neuritic plaques was also larger (Figs. 3C and 416.32 ± 103.90 µm^2^ in *Bace1*^*fl/fl*^;*Olig2-Cre; App*^*NL−G−F/wt*^ vs. 301.96 ± 97.87 µm^2^ in *Bace1*^*fl/fl*^;*App*^*NL−G−F/wt*^ mouse brain sections; *N* = 79 plaques in Bace1^fl/fl^;App^NL−G−F/wt^ and 80 in Bace1^fl/fl^;Olig2-Cre; App^NL−G−F/wt^ samples, *P* = 0.058). Similarly, the number of microglia in surrounding neuritic plaque was also higher in *Bace1*^*fl/fl*^;*Olig2-Cre; App*^*NL−G−F/wt*^ mouse brains (4.87 ± 0.73) compared to that in *Bace1*^*fl/fl*^;*App*^*NL−G−F/wt*^ (4.06 ± 0.41) (Fig. 3D-E, *P* = 0.032). However, the size of microglia appeared to not differ (Figs. 3F and 82.17 ± 19.80 µm^2^ in *Bace1*^*fl/fl*^;*App*^*NL−G−F/wt*^ vs. 85.14 ± 14.63 µm^2^ in *Bace1*^*fl/fl*^;*Olig2-Cre; App*^*NL−G−F/wt*^ brain sections; *N* = 79 plaques in Bace1^fl/fl^;App^NL−G−F/wt^ vs. 80 in Bace1^fl/fl^;Olig2-Cre; App^NL−G−F/wt^; *P* = 0.752). The number of dystrophic neurites, labeled by RTN3 antibody as previously discussed [50], was slightly less but not statistically significant (Supplemental Figure S2).

### Transcriptomic changes in response to the deletion of Bace1 in oligodendrocytes

**Fig. 4:**
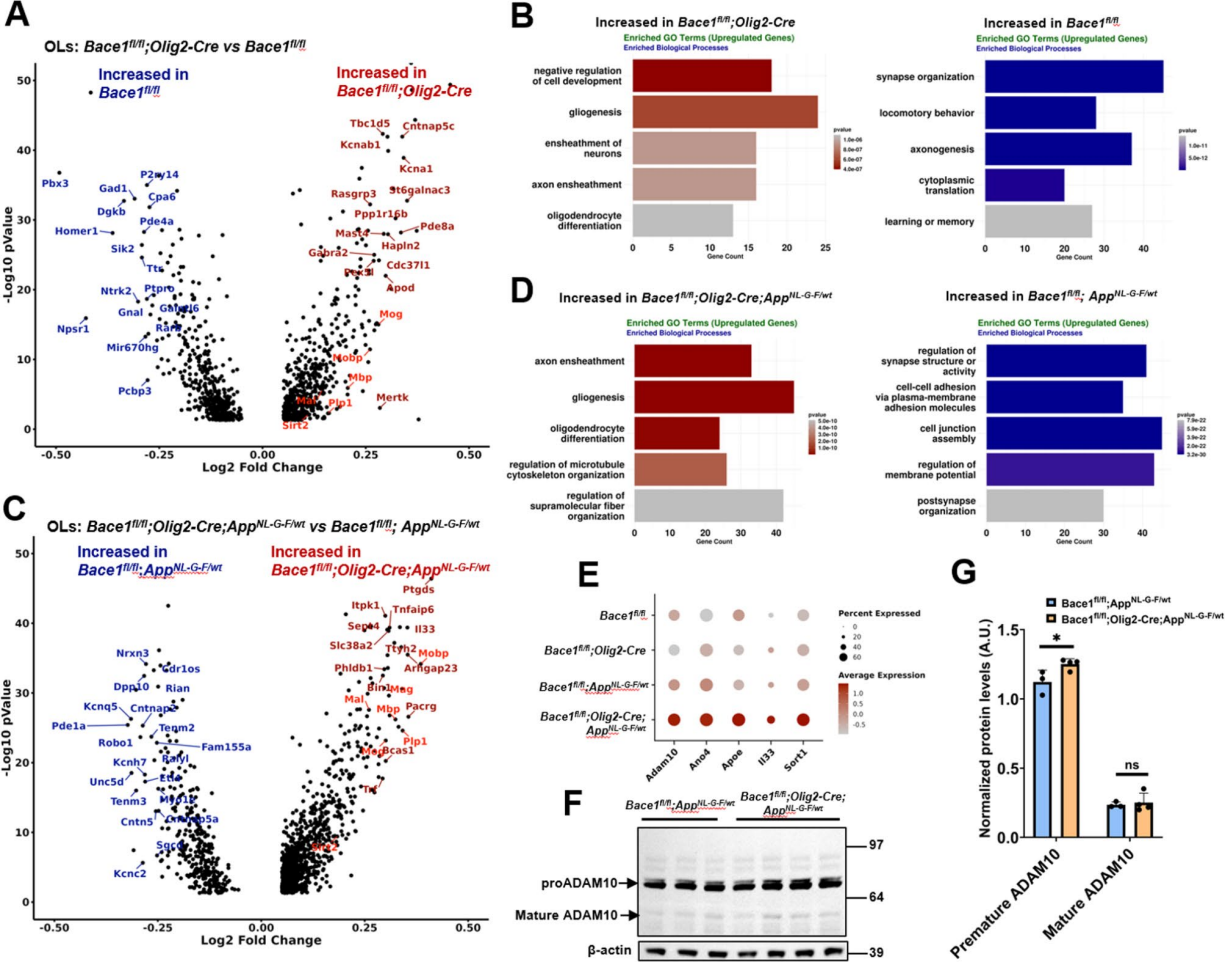
**Differential gene expression analysis and GO pathway analysis of OLs in*Bace1***^***fl/fl***^, ***Bace1***^***fl/fl***^;***Olig2-Cre***, ***Bace1***^***fl/fl***^;***Olig2-Cre; App***^***NL−G−F/wt***^**and*Bace1***^***fl/fl***^;***App***^***NL−G−F/wt***^**mice.** (**A**, **C**) Volcano plot depicting myelin genes labeled in red were differentially expressed in the oligodendrocytes of *Bace1*^*fl/fl*^;*Olig2-Cre* compared to *Bace1*^*fl/fl*^ mice in (**A**) and *Bace1*^*fl/fl*^;*Olig2-Cre; App*^*NL−G−F/wt*^ mice compared to *Bace1*^*fl/fl*^;*App*^*NL−G/wt*^ mice in (**C**). (**B**, **D**) GO biological process enrichment analyses of up-regulated and down-regulated genes in the oligodendrocytes of *Bace1*^*fl/fl*^;*Olig2-Cre* mice compared to *Bace1*^*fl/fl*^ mice in (B) and of *Bace1*^*fl/fl*^;*Olig2-Cre; App*^*NL−G−F/wt*^ mice compared to *Bace1*^*fl/fl*^;*App*^*NL−G/wt*^ mice in (**D**). (**E**) Dot plots show that the average gene expression of *ADAM10*, *Ano4*, *ApoE*, *Il33*, and *Sort1* increased in the oligodendrocytes of *Bace1*^*fl/fl*^;*Olig2-Cre; App*^*NL−G−F/wt*^ mice compared to other genotypes. (**F**-**G**) Immunoblot and quantification showing the level of ADAM10 increased in *Bace1*^*fl/fl*^;*Olig2-Cre; App*^*NL−G−F/wt*^ mice compared to *Bace1*^*fl/fl*^;*App*^*NL−G−F/wt*^ mice. *N* = 3 independent experiments, **P < 0.01, two-tailed Student t-test

Since plaque load was significantly reduced in the cerebral cortex and hippocampus in *Bace1*^*fl/fl*^;*Olig2-Cre; App*^*NL−G−F/wt*^ mice, we determined the molecular changes transcriptionally occurring in oligodendrocytes with *Bace1* deletion in the *App*^*NL−G−F/wt*^ mice. By performing unbiased snRNA-Seq using nuclei samples isolated from the cortex and hippocampus of ~ 5-month-old female mice from four groups (*Bace1*^*fl/fl*^, *Bace1*^*fl/fl*^;*Olig2-Cre*,* Bace1*^*fl/fl*^;*App*^*NL−G−F/wt*^, and *Bace1*^*fl/fl*^;*Olig2-Cre; App*^*NL−G−F/wt*^ mice), we were able to divide the nuclei into 37 clusters based on cell types clustered via the marker genes such as excitatory neurons (*Slc17a7*), inhibitory neurons (*Gad2*), astrocytes (*Aqp4*,* Clu*), microglia (*Cx3cr1*,* Hexb*), endothelial (*Cldn5*,* Vtn*), macrophage (*Marc1*), pericytes (*Atp13a5*), ependymal (*Kl*), OPC (*Pdgfra*) and OLs (*Mog*) (Supplemental Figure S3A-D). UMAP visualization of cellular populations in each genotype group did not reveal an obvious change when all cellular populations were included (Supplemental Figure S3E). We then focused only on oligodendrocytes as Bace1 was deleted mainly in this cell population. We identified the differentially expressed genes in oligodendrocytes between: (1) *Bace1*^*fl/fl*^;*Olig2-Cre* mice and *Bace1*^*fl/fl*^ mice, (2) *Bace1*^*fl/fl*^;*App*^*NL−G−F/wt*^ mice and *Bace1*^*fl/fl*^ mice, (3) *Bace1*^*fl/fl*^;*Olig2-Cre; App*^*NL−G−F/wt*^ mice and *Bace1*^*fl/fl*^;*App*^*NL−G−F/wt*^ mice, and (4) *Bace1*^*fl/fl*^;*Olig2-Cre; App*^*NL−G−F/wt*^ mice and *Bace1*^*fl/fl*^ mice (see volcano plots in Fig. 4A and B; Supplemental Table 1). When *Bace1* was deleted in oligodendrocytes, a modest increased expression of myelin genes such as *Mbp*, *Plp1*, *Mog*, *Mal*, *Sirt2* (marked in red in Fig. 4A and B) were commonly seen regardless of whether it was in WT (*Bace1*^*fl/fl*^;*Olig2-Cre* vs. *Bace1*^*fl/fl*^) or AD (*Bace1*^*fl/fl*^;*Olig2-Cre; App*^*NL−G−F/wt*^ vs. *Bace1*^*fl/fl*^;*App*^*NL−G−F/wt*^) mouse background, indicating that BACE1 regulates expression of these myelin genes. Elevation of these myelin genes was also seen in *Bace1*^*fl/fl*^;*Olig2-Cre; App*^*NL−G−F/wt*^ vs. *Bace1*^*fl/fl*^ mice (Supplemental Figure S4C). The differentially expressed genes in oligodendrocytes were further analyzed by Gene Ontology (GO) gene set enrichment analysis (GSEA) (Fig. 4C and D; Supplemental Figure S4B, D). The GO enrichment analysis showed that pathways for axon ensheathment, gliogenesis, and oligodendrocyte differentiation were upregulated in oligodendrocytes with *Bace1* deletion irrespective of presence of *App*^*NL−G−F*^ gene (Fig. 4C and D, Supplemental Figure S4B, 4D, Supplemental Table 1). Interestingly, pathways associated with synapse organization, axonogenesis, regulation of synapse organization, regulation of synapse structure or activity were reduced in the oligodendrocytes from both *Bace1*^*fl/fl*^;*Olig2-Cre* mice and *Bace1*^*fl/fl*^;*Olig2-Cre; App*^*NL−G−F/wt*^ mice when compared to *Bace1*^*fl/fl*^ mice (Fig. 4C and D, Supplemental Figure S4B, D, Supplemental Table 1).

Strikingly, several genes involved in Aβ production or clearance such as *Adam10*,* Ano4*, *ApoE*, *Il33*, *Sort1*, and *Sort1* were increased in the oligodendrocytes of *Bace1*^*fl/fl*^;*Olig2-Cre; App*^*NL−G−F/wt*^ mice when compared to *the other three groups of* mice (Fig. 4E). Since ADAM10 can function as α-secretase to mediate production of CTF83, we prepared the cortex lysate from 1-year-old *Bace1*^*fl/fl*^;*Olig2-Cre; App*^*NL−G−F/wt*^ mice and *Bace1*^*fl/fl*^;*App*^*NL−G−F/wt*^ mice and performed the immunoblot of ADAM10. We observed a significant increase of pro-ADAM10 levels in *Bace1*^*fl/fl*^;*Olig2-Cre; App*^*NL−G−F/wt*^ mice compared to *Bace1*^*fl/fl*^;*App*^*NL−G−F/wt*^ mice while mature ADAM10 was slightly elevated (Fig. 4F and G). The slight increase in ADAM10 protein levels may also contribute to the reduction of Aβ in the Bace1-deleted APP knock-in mice.

**Fig. 5:**
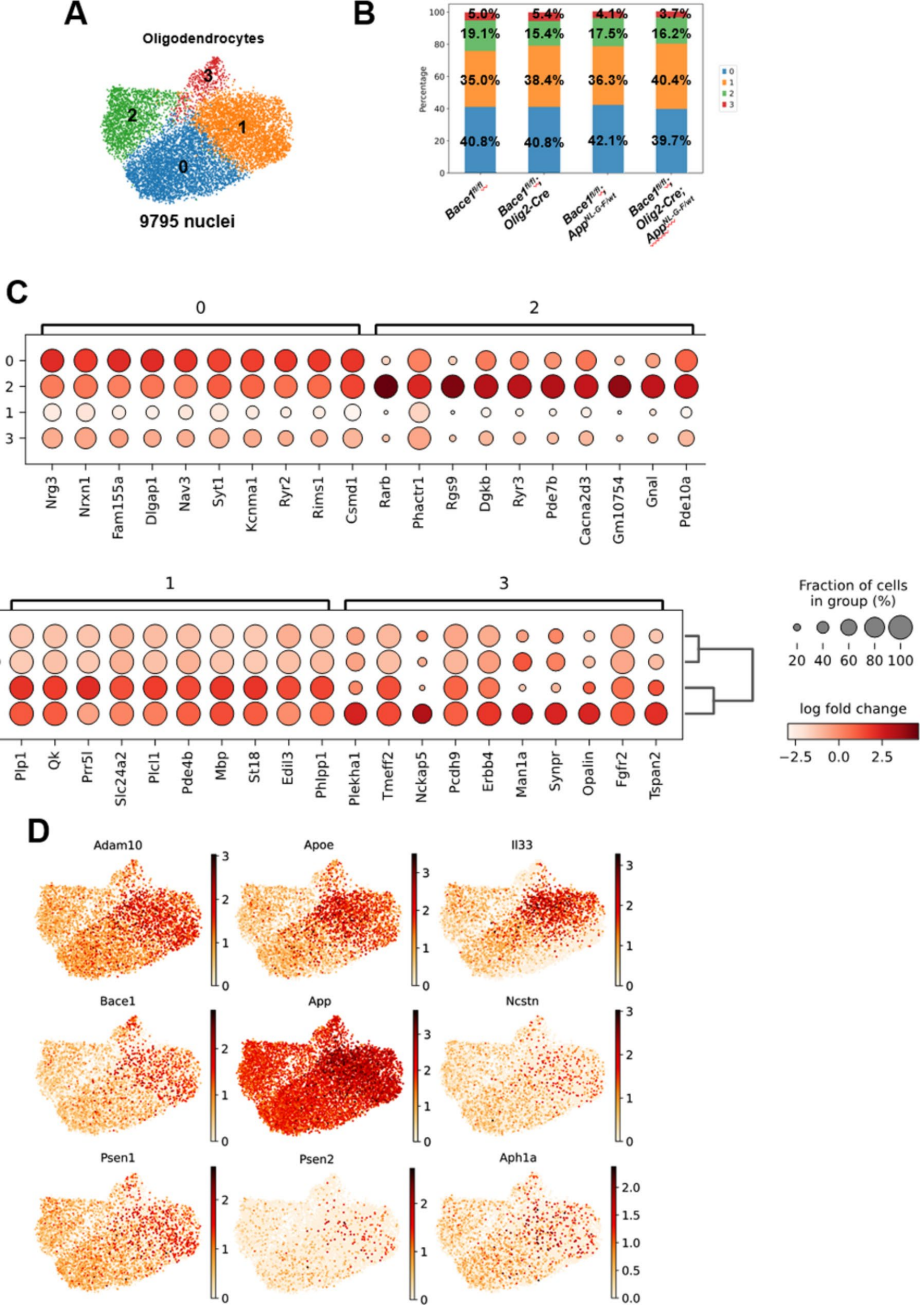
**OLs divided into four distinct clusters.** (**A**) UMAP visualization showing the oligodendrocytes nuclei from all genotypes grouped into four clusters. (**B**) The composition of cell percentages in each cluster across all genotypes remains comparable. (**C**) Dot plots show that the cluster markers were distinctly expressed in each cluster. (**D**) UMAP indicates that oligodendrocytes in cluster 1 exhibit relatively higher expression levels of *ADAM10*, *ApoE*, *Il33*, *Bace1*, *App*, *Ncstn*, *Psen1*, *Psen2*, and *Aph1a* compared to other clusters

Recent single-cell analysis identified four subclusters of oligodendrocytes in AD mice [28]. We pooled a total 9795 nuclei of oligodendrocytes to perform sub-clustering of oligodendrocyte nuclei and identified 4 subclusters based on specific marker genes (Fig. 5A-C). Interestingly, *Bace1* deletion or APP mutations in the knock-in mice did not cause significant changes in the cellular composition or proportion in each cluster (Fig. 5B). Compared to other clusters, cells in the cluster 1 express higher levels of genes involved in Aβ production or clearance or critical components of the gamma-secretase complex. This list of genes included *ADAM10*,* Apoe*,* Il33*,* Bace1*,* App*,* Ncstn*,* Psen1*,* Psen2*, and *Aph1a* (Fig. 5D), suggesting that cells in this cluster are likely the major source of oligodendrocyte Aβ.

### Oligodendrocyte *Bace1* deletion has a differential effect on the central myelination

**Fig. 6:**
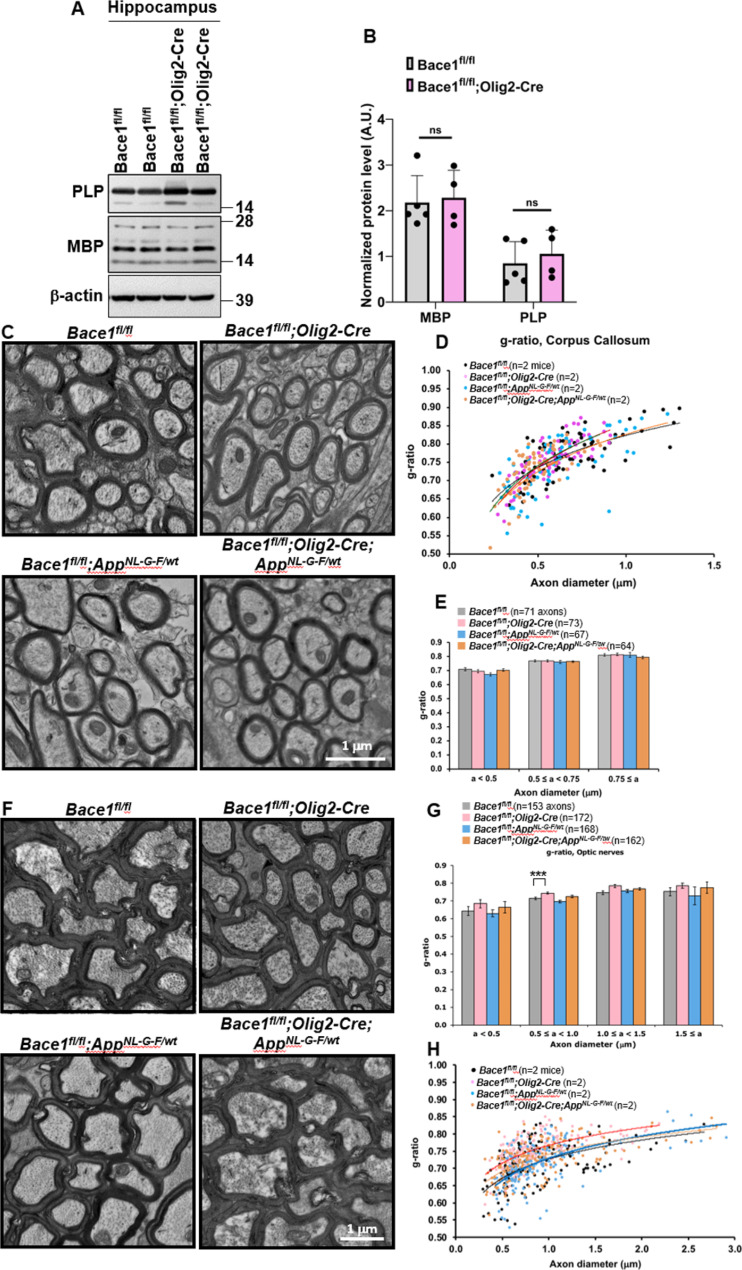
**Oligodendrocyte *Bace1 *deletion has differential effects on central or peripheral myelination.** (A) Immunoblot analysis of PLP and MBP in 4-month-old *Bace1*^*fl/fl*^ and *Bace1*^*fl/fl*^;*Olig2-Cre* hippocampi. Antibody to β-actin was used as loading control. (B) Bar graph shows quantification of relative protein levels based on the blot shown in A. *N* = 3 independent experiments, two-tailed Student’s *t* test. Values are expressed as mean ± SD. (C-E) Representative images of myelinated axons (C) in the corpus callosum and the scatter plot (D, *n* = 2 mice) as well as bar graph (E) against axonal diameter of the g-ratios of myelinated axons in all four groups. (F-H) Representative images (F) of myelinated axons in the optic nerves and bar graph (G) against axonal diameter of the g-ratios of myelinated axons in all four groups. The scatter plot is also presented (H, *N* = 2 mice in each genotype). Scale bar, 1 μm; axonal numbers are indicated in parenthesis; ***P < 0.001

Since global germline or neuronal *Bace1* deletion causes hypomyelination in the PNS [24, 60] and impaired maturation of oligodendrocytes [5, 24], we examined whether the loss of *Bace1* from oligodendrocytes would affect levels of myelin proteins and the thickness of myelin sheath. The above snRNA-Seq results in Fig. 4A and B suggested an elevation of myelin gene expression. We isolated hippocampal protein lysates from 4-month-old *Bace1*^*fl/fl*^*and Bace1*^*fl/fl*^;*Olig2-Cre* mice and performed immunoblot experiments. We showed that MBP and PLP levels were not significantly elevated in *Bace1*^*fl/fl*^;*Olig2-Cre* mice compared to controls (Fig. 6A, B). Consistent with this, we found no significant change in myelin sheath thickness in corpus callosum axons from *Bace1*^*fl/fl*^, *Bace1*^*fl/fl*^;*Olig2-Cre*,* Bace1*^*fl/fl*^;*App*^*NL−G−F/wt*^, and *Bace1*^*fl/fl*^;*Olig2-Cre; App*^*NL−G−F/wt*^ mice at 4 months of age by EM analysis (Fig. 6C, g-ratio comparisons plotted in 6D-E). However, we found that the axons in the optic nerves were wrapped by thinner myelin sheaths in the *Bace1*^*fl/fl*^;*Olig2-Cre* mice compared with *Bace1*^*fl/fl*^ mice (Fig. 6G). Morphometric quantification of myelin thickness by g-ratio analysis (ratio of individual axon diameters to myelinated fiber diameters) in the optic nerve confirmed the relative decrease in myelin thickness (higher g-ratio shown in Fig. 6G). When *Bace1*^*fl/fl*^;*Olig2-Cre* mice were compared with littermate controls, the most significant differences were observed in axons ranging between 0.5 and 1.0 μm [average g-ratios: 0.71 ± 0.007 in *Bace1*^*fl/fl*^ (*N* = 69 axons) vs. 0.74 ± 0.005 in *Bace1*^*fl/fl*^;*Olig2-Cre* (*N* = 65 axons); *P* = 5.3 × 10^− 7^], but not the other size groups. However, this significance could not be calculated if only 2 mice per genotype group were used for analysis (Fig. 6H, *N* = 2). Future study will be needed for further validation. No discernible axonal degeneration was observed in the optic nerves and corpus callosum in all genotypes.

### *Bace1*^*fl/fl*^;*Olig2-Cre* mice exhibit normal hippocampal activity-dependent synaptic plasticity

**Fig. 7:**
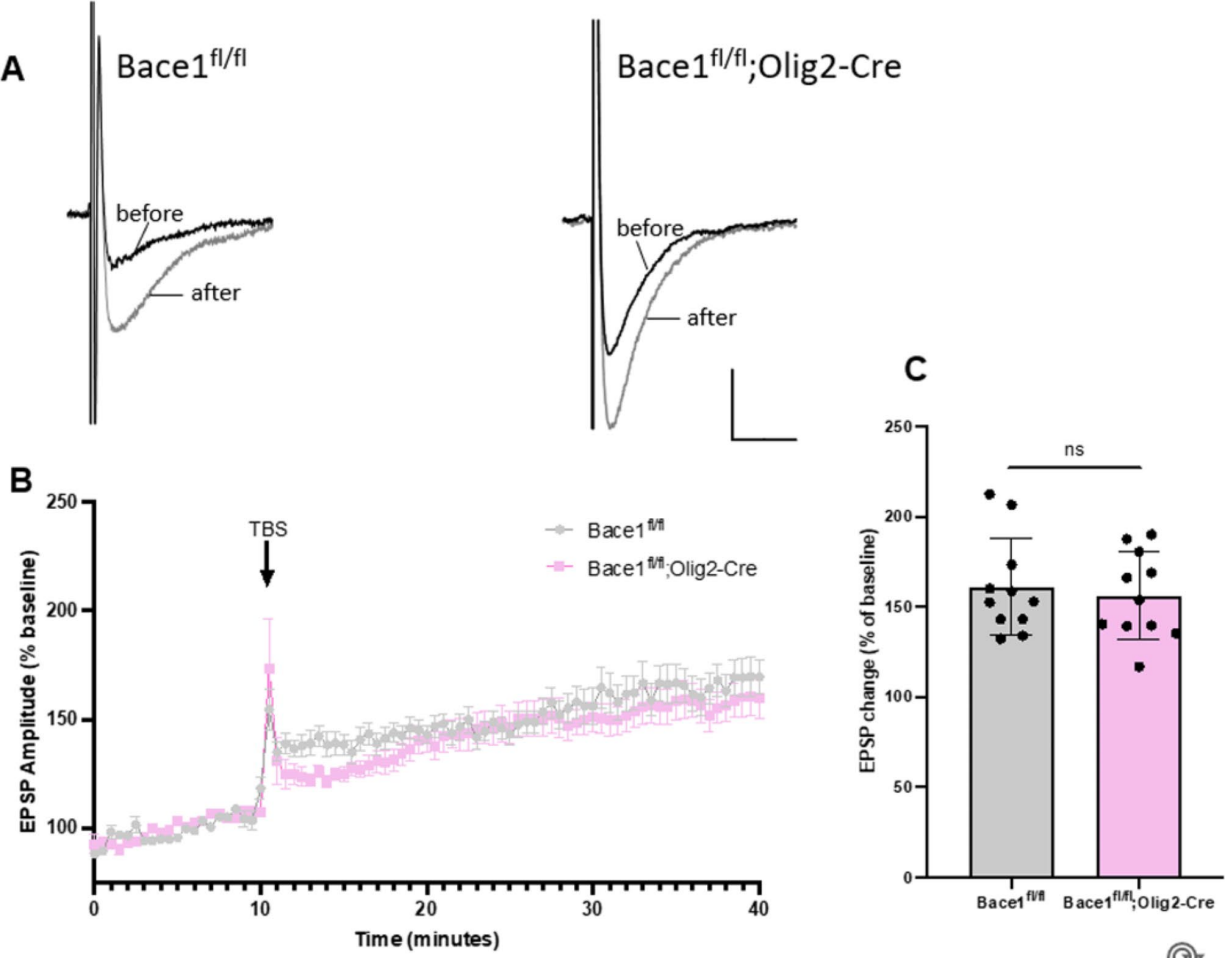
**No impairments in Synaptic Plasticity in*Bace1***^***fl/fl***^;***Olig2-Cre mouse brains***. (A) An overlay of the representative fEPSPs from the hippocampal Schaffer Collateral-CA1 synapse in ~ 4-months-old *Bace1*^*fl/fl*^ and *Bace1*^*fl/fl*^;*Olig2-Cre* mice brain slices, before (black trace), and after (gray trace) the induction of LTP. Calibration bars: 10 ms, 0.1 mV. (B) Plots of Theta-burst stimulation (TBS)-induced LTP in *Bace1*^*fl/fl*^ and *Bace1*^*fl/fl*^;*Olig2-Cre* hippocampal brain slices. Field EPSP Amplitude (% Baseline) over time is shown (*Bace1*^*fl/fl*^, *N* = 3 mice / 10 slices; *Bace1*^*fl/fl*^;*Olig2-Cre*, *N* = 3 mice / 12 slices, ). (C) Bar graph of field EPSP amplitude (% Baseline) 20 min after LTP induction. Error bars represent mean ± SD, n.s. = *p* > 0.05 (two-tailed t-test)

Deficits in synaptic plasticity have previously been reported in global and neuron-specific *Bace1* knockout models or BACE1 inhibition [9, 32, 37, 41]; Zhu et al., [22]). Our sequencing results showed reduction of genes in oligodendrocytes involved in synapse organization (Fig. 4C, D). To identify potential changes in synaptic strength associated with oligodendrocyte *Bace1* deletion, we examined activity-dependent synaptic plasticity in the hippocampus of 12-month-old *Bace1*^*fl/fl*^;*Olig2-Cre* mice and *Bace1*^*fl/fl*^ littermate controls using field potential recordings. Specifically, we measured field excitatory postsynaptic potentials (fEPSPs) from Schaffer collateral axons projecting to CA1 apical dendrites before and after the induction of LTP (Fig. 7A). The LTP magnitude was marginally decreased in *Bace1*^*fl/fl*^;*Olig2-Cre* mice when compared to *Bace1*^*fl/fl*^ littermates (Figs. 7B-C and 156.21 ± 7.26% in *Bace1*^*fl/fl*^;*Olig2-Cre vs.*. 160.8 ± 8.09% in *Bace1*^*fl/fl*^, *P* > 0.05). Hence, synaptic strength is unlikely affected if Bace1 is only deleted in oligodendrocytes.

## Discussion

BACE1 is essential for the generation of Aβ, a major component of AD pathology found in AD brains. Unfortunately, previous attempts at lowering BACE1 levels have largely failed to improve cognition in AD trials [35]. This is likely due to inhibition of neuronal BACE1, which cleaves SEZ6, neuregulin members, and other substrates important for normal neuronal functioning [2, 54]. This has prompted the need to identify alternative BACE1 targeting strategies. We found high expression levels of Bace1 in oligodendrocytes based on our single-cell results [51]. However, little is known about the contribution of oligodendrocyte BACE1 to amyloid plaque formation in AD or the side effects associated with oligodendrocyte-specific *Bace1* deletion. Here, we show that deletion of oligodendrocyte *Bace1* has negligible effects on myelination of the central nervous system and LTP reduction. Our most important finding is that oligodendrocyte *Bace1* deletion significantly reduces Aβ plaque load in an AD mouse model. This observation is in line with the recent publications that oligodendrocytes are active contributors to Aβ plaque burden in AD [42, 48]. Together, we highlight a previously underappreciated role of oligodendrocytes in AD pathology, suggesting that these cells might be key contributors to disease progression and a future explorable target for AD therapy.

In this study, we generated mice with conditional oligodendrocyte-specific *Bace1* deletion (*Bace1*^*fl/fl*^;*Olig2-Cre*). These mice lacked *Bace1* mRNA in *Mbp*^*+*^ oligodendrocytes, whereas *Syn*^*+*^ neuronal *Bace1* was unaffected. We found that overall BACE1 and APP-fl protein levels in *Bace1*^*fl/fl*^;*Olig2-Cre* hippocampal tissues were comparable to those of Bace1^fl/fl^ controls, likely due to unaffected *Bace1* expression in neurons and other glial cells that masked reduction of Bace1 in oligodendrocytes. This was confirmed in isolated O4 + oligodendrocytes which, showed a significant reduction in BACE1 levels and elevated levels of full-length APP, indicative of decreased APP cleavage and consistent with loss of function of BACE limited to oligodendrocytes.

We examined the impact of *Bace1* deletion on cellular function of oligodendrocytes. Previous studies have shown that neuron-specific *Bace1* impairs maturation of oligodendrocytes [5] and reduces hippocampal MBP and PLP levels [24]. Unbiased sequencing results showed that myelin genes such as Mbp, Mog, Mag, and Plp were elevated when *Bace1* was deleted in oligodendrocytes (Fig. 4A and B), an observation contrary to the deletion of *Bace1* in neurons or germline deletion of Bace1. These results indicate that conditional loss of function of BACE1 in mature oligodendrocytes may lead to a compensatory upregulation of remyelination programs. These results suggest that BACE1 in oligodendrocytes and neurons governs differential signaling pathways that control myelin gene expression. In hippocampal neurons, type III neuregulin-1 was speculated to be the BACE1 substrate to control myelination [16, 23]. We do not yet know the oligodendrocyte BACE1 substrate that will control the expression of many myelin genes. We found no alterations in myelin sheath thickness of corpus collosum axons between *Bace1*^*fl/fl*^;*Olig2-Cre* and *Bace1*^*fl/fl*^ mice, despite a small increase in the expression of many myelin genes. One interesting observation is the slight reduction in myelin sheath thickness in medium-sized (0.5 to 1.0 μm) optic nerves (Fig. 6F-G), and this reduction was seen in *Bace1*-null mouse optic nerves [24]. This result implies that signaling pathways in the optic nerve and corpus collosum nerves are differentially regulated. Since myelin genes are upregulated during the peak of myelination and then gradually reduce during adulthood [18], the increase of myelin genes and myelination pathways in *Bace1*^*fl/fl*^;*Olig2-Cre* mice and *Bace1*^*fl/fl*^;*Olig2-Cre; App*^*NL−G−F/wt*^ mice relative to controls without Bace1 deletion is likely due to a delay in the reduction of myelin genes in the absence of Bace1 in oligodendrocytes during adulthood. Future additional biochemical studies may be warranted to reveal such differences in the control of central myelination. One encouraging observation is that we saw no significant reduction in LTP measured from the Schaffer Collateral-CA1 synapses in *Bace1*^*fl/fl*^;*Olig2-Cre* mice compared to *Bace1*^*fl/fl*^ littermates (Fig. 7), unlike global or neuronal deletion of Bace1 in mice, which exhibit reduction in synaptogenesis and LTP reduction [5].

To investigate the role of BACE1 in oligodendrocytes, we conducted unbiased snRNA-Seq experiments to explore the underlying mechanism associated with amyloid plaque reduction. Our analysis using unbiased snRNA-Seq revealed that expression of *ADAM10*, *Ano4*, IL-*33*, *ApoE*, and *Sort1* were elevated in the oligodendrocytes of *Bace1*^*fl/fl*^;*Olig2-Cre; App*^*NL−G−F/wt*^ mice compared to other genotypes (Fig. 4E). The observed increase in ADAM10 was surprising as it is an α-secretase known to cleave APP within the Aβ region to preclude Aβ formation. Ano4 modulates ADAM10 sheddase activity [30]. The increase of *ADAM10* and *Ano4* genes in heterozygous *App*^*NL−G−F/wt*^ mice with *Bace1* deletion in oligodendrocytes is expected to promote non-amyloidogenic processing to reduce Aβ generation. Although we did not see obvious changes in CTF83 levels in hippocampal lysates, CTF83 levels were elevated in isolated O4^+^-cells (Fig. 2A) and mirrored the reduction of CTF99.

The observed increases in ApoE expression are in accord with findings in multiple cell types upon deletion of Bace1 including astrocytes [67] and microglia [51, 52]. Although higher levels of ApoE are expected to facilitate Aβ clearance [14, 15, 34, 43, 59], pathological ApoE4 in human oligodendrocytes, paradoxically impairs myelination and lipid metabolism [6].

While the exact function of IL-33 in oligodendrocytes is not well understood, IL-33 is upregulated in response to injury and disease and is known to play a neuroprotective role. IL-33 administration into APP/PS1 mice has been shown to reduce soluble Aβ levels and amyloid plaque deposition by promoting the recruitment of microglia for enhancing Aβ phagocytic activity [17]. Interestingly, we noted more astrocytes and microglia in surrounding amyloid plaques (Fig. 3), and this increase may facilitate clearance of amyloid plaques. In addition, Sortilin1 (SORT1) has been shown to decrease Aβ levels by degrading APP through the interaction between the intracellular domain of SORT1 with APP [46]. Thus, the higher expression of *IL-33*, *ApoE*, and *Sort1*, in the absence of *Bace1* in oligodendrocytes with the presence of *App*^*NL−G−F*^, is also speculated to contribute to the clearance of Aβ in the *Bace1*^*fl/fl*^;*Olig2-Cre; App*^*NL−G−F/wt*^ mice.

In APP-KI heterozygous mice, we noted a group of upregulated genes such as *Lrrtm3* (leucine rich repeat transmembrane neuronal 3), *Gphn* (gephyrin), and Nrp1 (neuropilin 1) (Supplemental Table 2). These genes have known associations with late-onset AD, amyloid plaques, and severe AD pathology. LRRTM3 is a late-onset AD gene, and its elevation appears to promote BACE1-mediated cleavage of APP [33]. Gephyrin accumulations in AD overlap with amyloid plaques but not with neurofibrillary tangles [1]. NRP1 is a transmembrane protein regulating mitochondrial function and iron homeostasis and was recently discovered to be elevated in people with severe AD [7, 31]. Our unbiased sequencing results from 5-month-old heterozygous APP-KI mice, which haven’t yet developed amyloid plaques, showed elevated expression of these AD-related genes. Remarkably, deleting *Bace1* appeared to reverse this elevation, suggesting a potential role in modulating early disease processes. This could mean BACE1 inhibition might have a broader impact on AD pathology than previously thought. (Supplemental Table 2).

In summary, our results, along with two recent publications [42, 48], provide direct evidence that Aβ generation is not limited to neuronal sources, but that oligodendrocytes are also important contributors to Aβ pathology within the AD brain. One intriguing observation is that *Bace1* deletion in excitatory neurons leads to a 95–98% reduction in amyloid burden [48] or near elimination of plaques in *Thy1*-positive neuron *Bace1*-KO mice [42], despite presence of *Bace1* in oligodendrocytes, which contribute 25–30% of amyloid plaques. One possible explanation is that neuronal Aβ may initiate seeding of amyloid plaques due to a relatively higher Aβ levels, while oligodendrocyte-derived Aβ perhaps mainly promotes plaque growth. One should note that these two studies used relatively young animals. The growth of amyloid plaques depends on various conditions including the aging component. Without neuronal Aβ, aggregation might take much longer. Examining older APP KI mice could confirm if plaques still form. Interestingly, suppressing oligodendrocyte Aβ seems to rescue early neuronal dysfunction [42], highlighting a more expansive role in AD pathogenesis. Moreover, our transcriptomic analysis revealed significant changes in response to *Bace1* deletion in oligodendrocytes, highlighting both known and novel immune regulatory pathways, that are upregulated in response to pathological conditions. While oligodendrocyte involvement in AD and related disease (ADRD) has been emerging, it hasn’t gained much traction in the field. There remains a vast, untapped potential in exploring this cell type’s role in disease progression as discussed in a recent perspective [44].

## Electronic supplementary material

Below is the link to the electronic supplementary material.

Supplementary Material 1

Supplementary Material 2

Supplementary Material 3

Supplementary Material 4

## Data Availability

All original data presented in the paper will be made available for reviews when needed. Research materials will be also made available when it is required.
