# Supplementary material for: Contribution of amyloid deposition from oligodendrocytes in a mouse model of Alzheimer’s disease
Source: Mol Neurodegener. 2024 Nov 16;19:83. doi: 10.1186/s13024-024-00759-z (PMC11568619; doi:10.1186/s13024-024-00759-z)
Supplement: Supplementary file 3 — Supplementary Material 3 [file 13024_2024_759_MOESM3_ESM.doc]

**Supplemental Figures and legends**

**Contribution of amyloid deposition from oligodendrocytes in a mouse model of Alzheimer’s disease**

Akihiro Ishii^1*^, Joseph A. Pathoulas^1*^, Omar Moustafa Fathy Omar^2^, Yingying Ge^1^, Annie Y Yao^1^, Tressa Pantalena^1^, Neeraj Singh^1^, John Zhou^1^, Wanxia He^1^, Patrick Murphy^2^, Riqiang Yan^1#^ and Xiangyou Hu^1#^,

^1^Department of Neuroscience, ^2^Department of Cell Biology and Vascular Biology Center, University of Connecticut Health Center,
 263 Farmington Avenue, Farmington, USA CT 06030-3401

^*^Both authors are contributed equally to this study.

^#^Xiangyou Hu, Ph.D.

University of Connecticut Health Center

Department of Neuroscience

263 Farmington Avenue

Farmington, CT USA 06030-3401

[xhu@uchc.edu](mailto:xhu@uchc.edu)

Or Riqiang Yan, Ph.D.

[riyan@uchc.edu](mailto:riyan@uchc.edu)

Tel 860-679-3527

Fax: 860-679-8766

Running title: *Bace1* deficiency in oligodendrocytes reducing plaque load

- ***Authors' information***

Ishii,Akihiro [ishii@uchc.edu](mailto:ishii@uchc.edu)

Pathoulas,Joseph Andrew [jpathoulas@uchc.edu](mailto:jpathoulas@uchc.edu)

Omar,Omar Moustafa Fathy [oomar@uchc.edu](mailto:oomar@uchc.edu)

Ge,Yingying [yge@uchc.edu](mailto:yge@uchc.edu)

Yao,Annie ayao@uchc.edu

Pantalena, Tressa M. [tressa1930@gmail.com](mailto:tressa1930@gmail.com)

Singh, Neeraj [nsingh@uchc.edu](mailto:nsingh@uchc.edu)

Zhou,John [jzhou@uchc.edu](mailto:jzhou@uchc.edu)

He,Wanxia, [wahe@uchc.edu](mailto:wahe@uchc.edu)

Murphy,Patrick [pamurphy@uchc.edu](mailto:pamurphy@uchc.edu)

Hu,Xiangyou [xhu@uchc.edu](mailto:xhu@uchc.edu)


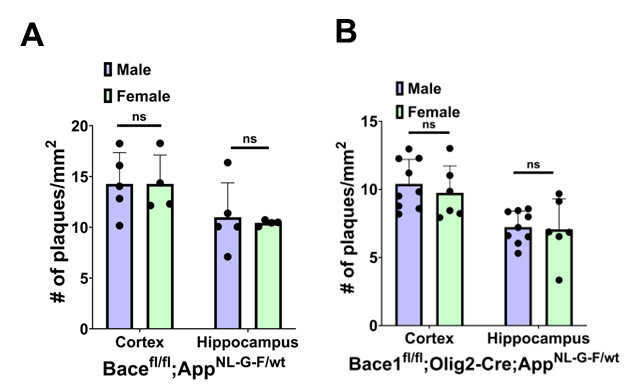


**Supplemental Figure S1: No gender effect of Bace1 deletion in oligodendrocytes in AD mice.** Sex comparison of plaque load in *Bace1^fl/fl^; App^NL-G-F/wt^* (A) and *Bace1^fl/fl^;Olig2-Cre;App^NL-G-F/wt^* mice (B) were not differed, indicating that Bace1 deletion has no gender effect. *N* = 5 male and 4 female *Bace1^fl/fl^; App^NL-G-F/wt^* mice, and 9 male and 6 female *Bace1^fl/fl^;Olig2-Cre;App^NL-G-F/wt^*; five sections were selected in every 10^th^ per mouse. Each dot represents one mouse.


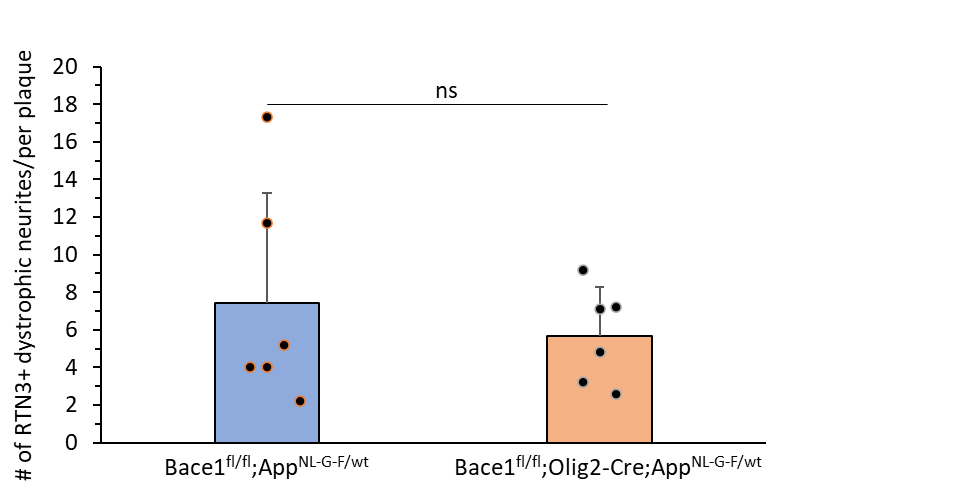


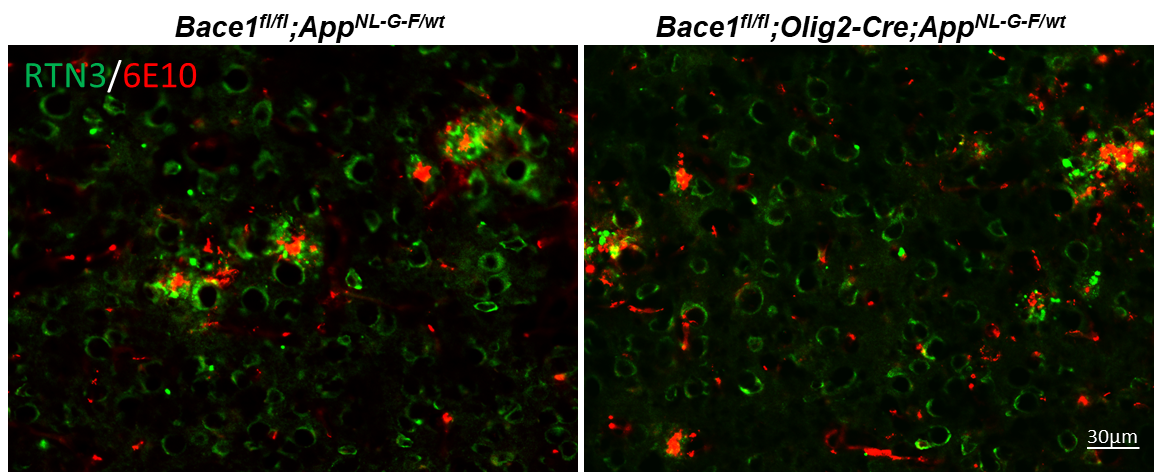
**Supplemental Figure S2: Dystrophic neurites marked by reticulon 3 antibody were slightly reduced in Bace1 deleted AD mice.** Number of RTN3+ dystrophic neurites surrounding neuritic plaque was 7.40 ± 5.86 in *Bace1^fl/fl^;App^NL-G-F/wt^* compared to 5.68 ± 2.57 in *Bace1^fl/fl^;Olig2-Cre;App^NL-G-F/wt^* mouse brain sections (A total of 41 plaques in *Bace1^fl/fl^;App^NL-G-F/wt^* while 70 plaques in *Bace1^fl/fl^;Olig2-Cre;App^NL-G-F/wt^* brain sections was counted). Each dot represents one mouse. Error bars represent mean ± SD, n.s. = p > 0.05 (two-tailed Student t-test).


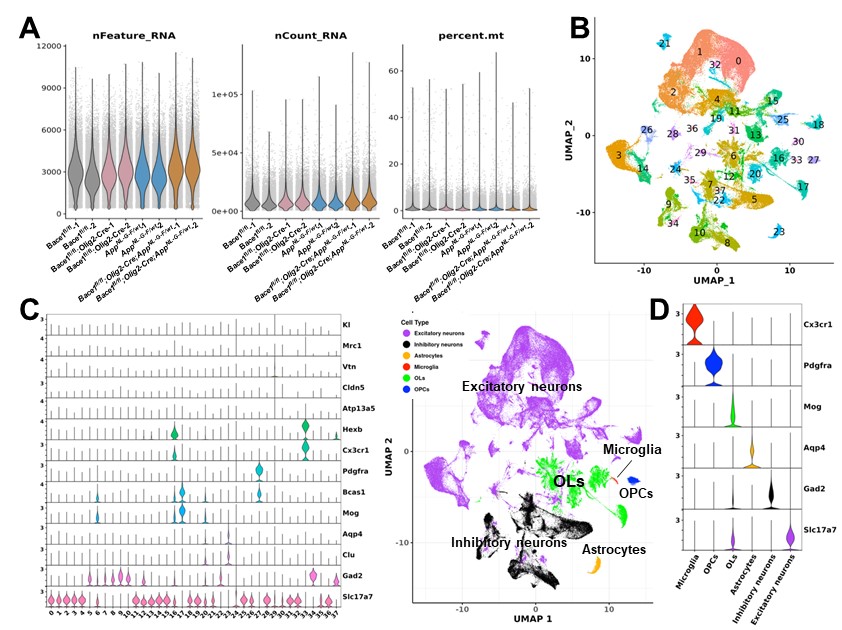


**
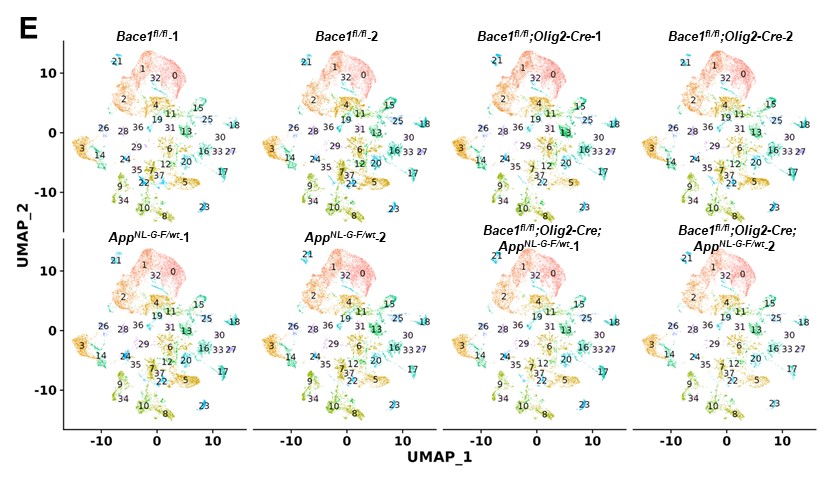
Supplemental Figure S3. Single nuclei RNA sequencing analysis.** (A) Violin plots showing the number of detected unique genes (“nFeature_RNA”), the number of unique molecular identifiers (UMIs, “nCount_RNA”), and the percentage of mitochondrial genes (“percent.mt”) in each nucleus from each sample before filtration. (B) UMAP visualization showing the unbiased assignment of nuclei to 37 clusters. (C) Six main cell types assigned to the UMAP clusters based on canonical markers. (D) Canonical cell type markers denote clusters of cell type as identities. (E) UMAP clusters of single nuclear sequencing from eight mouse cortex and hippocampi indicate that the genetic differences between these genotypes may not have a dramatic impact on the overall cellular populations.

**
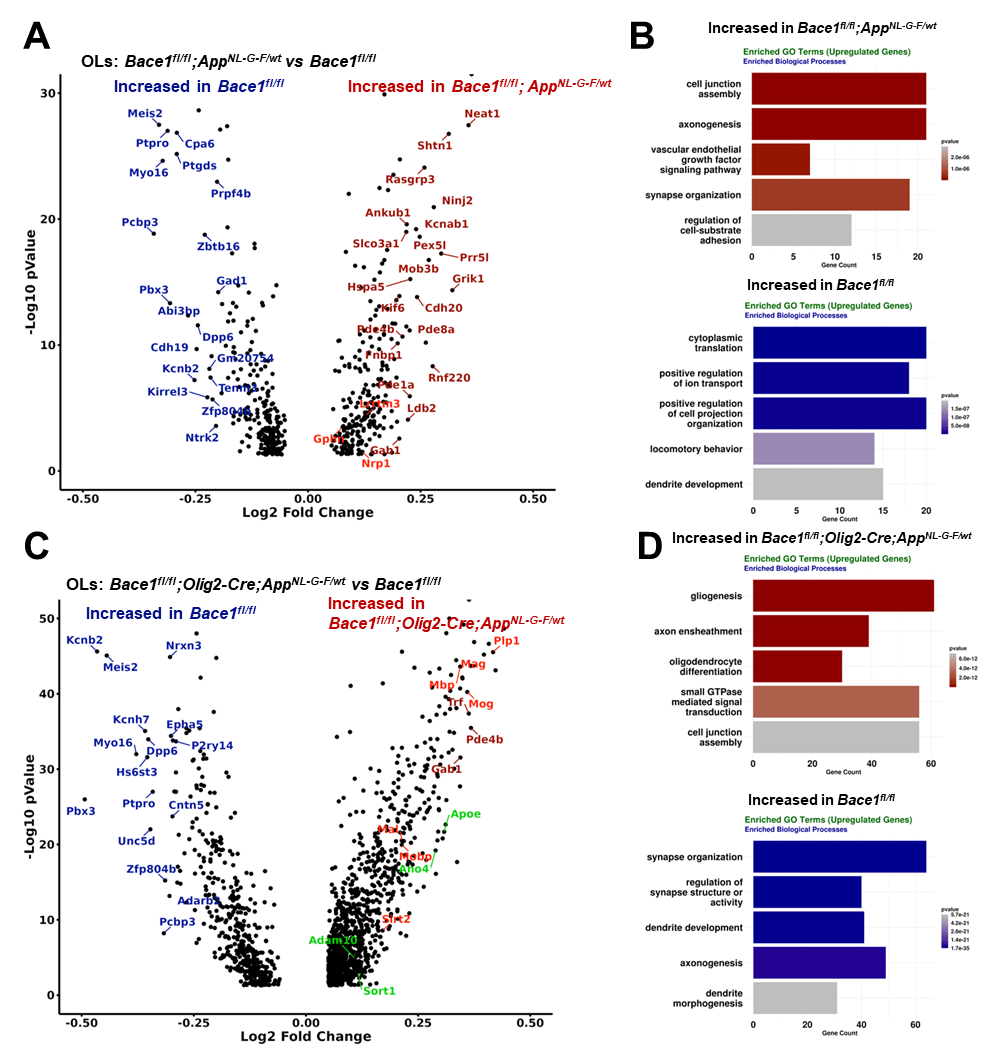
**

**Supplement Figure 4. Differential gene expression analysis and GO pathway analysis of OLs in *Bace1^fl/fl^*, *Bace1^fl/fl^;App^NL-G-F/wt^* mice, and *Bace1^fl/fl^;Olig2-Cre;App^NL-G-F/wt^* mice.** (A) Volcano plot depicting differentially expressed genes in the oligodendrocytes of *Bace1^fl/fl^;App^NL-G-F/wt^* mice compared to *Bace1^fl/fl^* mice. (B) GO biological process enrichment analyses of up-regulated and down-regulated genes in the oligodendrocytes of *Bace1^fl/fl^;App^NL-G-F/wt^* mice compared to *Bace1^fl/fl^* mice. (C) Volcano plot showing the differentially expressed gene in the oligodendrocytes between *Bace1^fl/fl^;Olig2-Cre;App^NL-G-F/wt^* mice and *Bace1^fl/fl^* mice. Myelin genes are labeled in red, and genes involved in the generation of sAPPα and the clearance of Aβ are labeled in green. (D) GO biological process enrichment analyses of up-regulated and down-regulated genes in the oligodendrocytes of *Bace1^fl/fl^;Olig2-Cre;App^NL-G-F/wt^* mice compared to *Bace1^fl/fl^* mice. Comparing the effect of the *App^NL-G-F^* knock-in in oligodendrocytes to the *Bace1^fl/fl^* control, we found that the pathways for cell junction assembly and axonogenesis were increased and the pathways for cytoplasmic translation, iron transport, and ribosomal small subunit biogenesis were decreased in the oligodendrocytes of *Bace1^fl/fl^;App^NL-G-F/wt^* mice.
