# Supplementary material for: Contribution of amyloid deposition from oligodendrocytes in a mouse model of Alzheimer’s disease
Source: Mol Neurodegener. 2024 Nov 16;19:83. doi: 10.1186/s13024-024-00759-z (PMC11568619; doi:10.1186/s13024-024-00759-z)
Supplement: Supplementary file 4 — Supplementary Material 4 [file 13024_2024_759_MOESM4_ESM.pptx]

## Slide 1
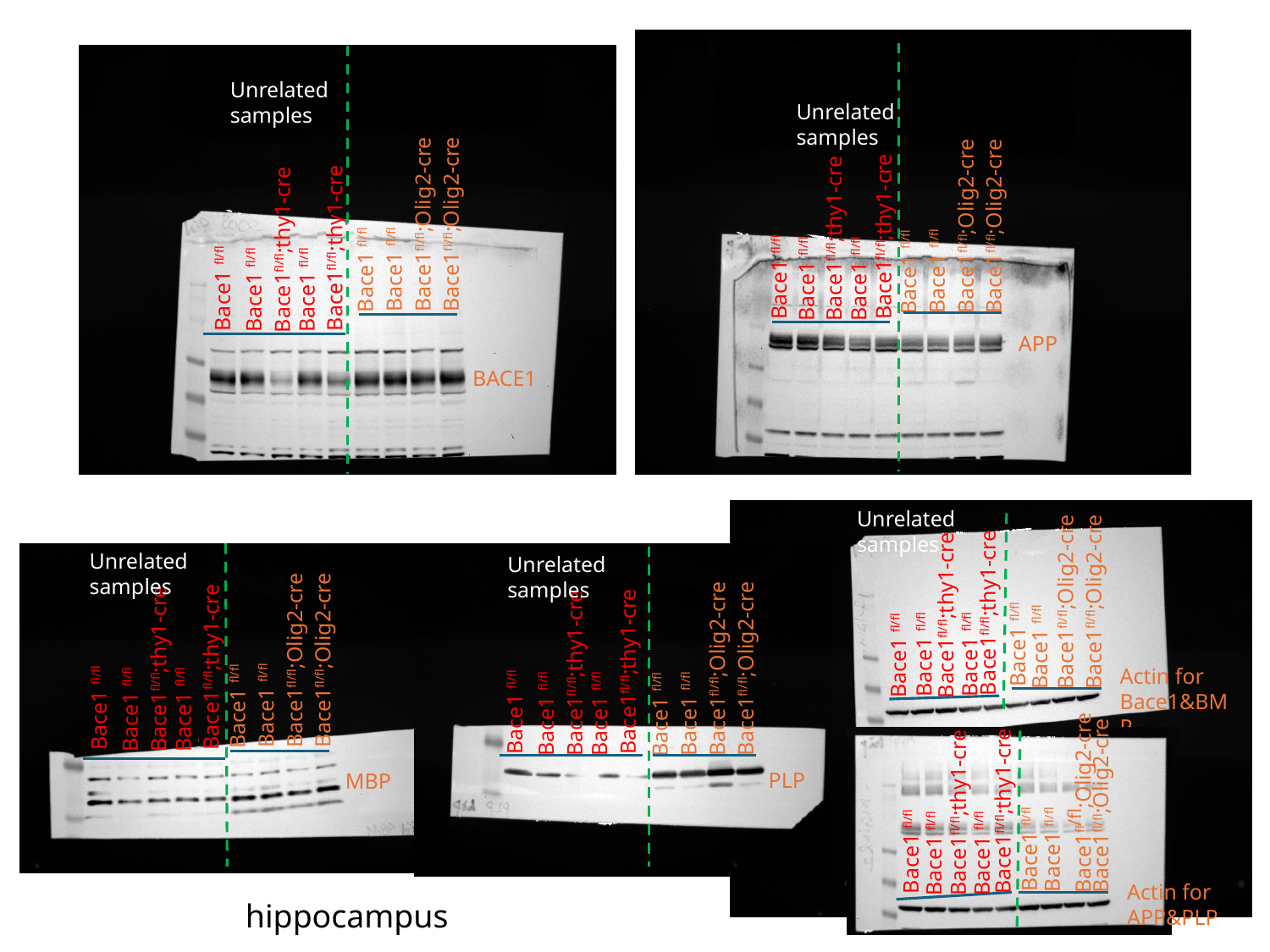

Unrelated samples
Unrelated samples
Bace1fl/fl;Olig2-cre
Bace1fl/fl;Olig2-cre
Bace1fl/fl;Olig2-cre
Bace1fl/fl;Olig2-cre
Bace1fl/fl;thy1-cre
Bace1fl/fl;thy1-cre
Bace1fl/fl;thy1-cre
Bace1fl/fl;thy1-cre
Bace1 fl/fl
Bace1 fl/fl
Bace1 fl/fl
Bace1 fl/fl
Bace1 fl/fl
Bace1 fl/fl
Bace1 fl/fl
Bace1 fl/fl
Bace1 fl/fl
Bace1 fl/fl
APP
BACE1
Unrelated samples
Bace1fl/fl;Olig2-cre
Bace1fl/fl;Olig2-cre
Unrelated samples
Unrelated samples
Bace1fl/fl;thy1-cre
Bace1fl/fl;thy1-cre
Bace1fl/fl;Olig2-cre
Bace1fl/fl;Olig2-cre
Bace1 fl/fl
Bace1fl/fl;Olig2-cre
Bace1fl/fl;Olig2-cre
Bace1 fl/fl
Bace1 fl/fl
Bace1 fl/fl
Bace1 fl/fl
Bace1fl/fl;thy1-cre
Bace1fl/fl;thy1-cre
Bace1fl/fl;thy1-cre
Bace1fl/fl;thy1-cre
Actin for Bace1&BMP
Bace1 fl/fl
Bace1 fl/fl
Bace1 fl/fl
Bace1 fl/fl
Bace1 fl/fl
Bace1 fl/fl
Bace1 fl/fl
Bace1 fl/fl
Bace1 fl/fl
Bace1 fl/fl
Bace1fl/fl;Olig2-cre
Bace1fl/fl;Olig2-cre
PLP
MBP
Bace1fl/fl;thy1-cre
Bace1fl/fl;thy1-cre
Bace1 fl/fl
Bace1 fl/fl
Bace1 fl/fl
Bace1 fl/fl
Bace1 fl/fl
Actin for APP&PLP
hippocampus

## Slide 2
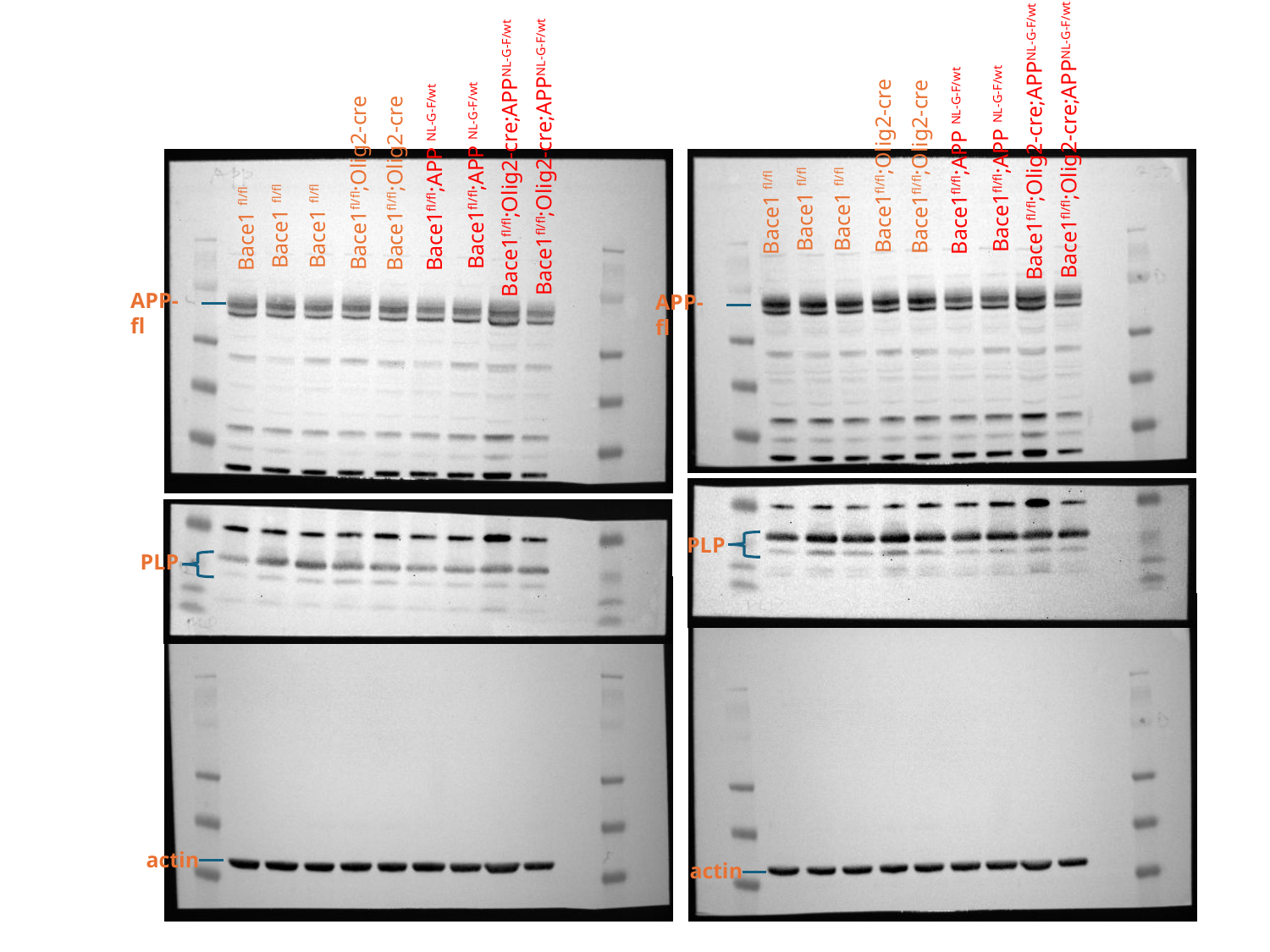

Bace1fl/fl;Olig2-cre;APPNL-G-F/wt
Bace1fl/fl;Olig2-cre;APPNL-G-F/wt
Bace1fl/fl;Olig2-cre
Bace1fl/fl;Olig2-cre
Bace1fl/fl;APP NL-G-F/wt
Bace1fl/fl;Olig2-cre;APPNL-G-F/wt
Bace1fl/fl;APP NL-G-F/wt
Bace1fl/fl;Olig2-cre;APPNL-G-F/wt
Bace1fl/fl;Olig2-cre
Bace1fl/fl;Olig2-cre
Bace1fl/fl;APP NL-G-F/wt
Bace1fl/fl;APP NL-G-F/wt
Bace1 fl/fl
Bace1 fl/fl
Bace1 fl/fl
Bace1 fl/fl
Bace1 fl/fl
Bace1 fl/fl
APP-fl
APP-fl
PLP
PLP
actin
actin

## Slide 3
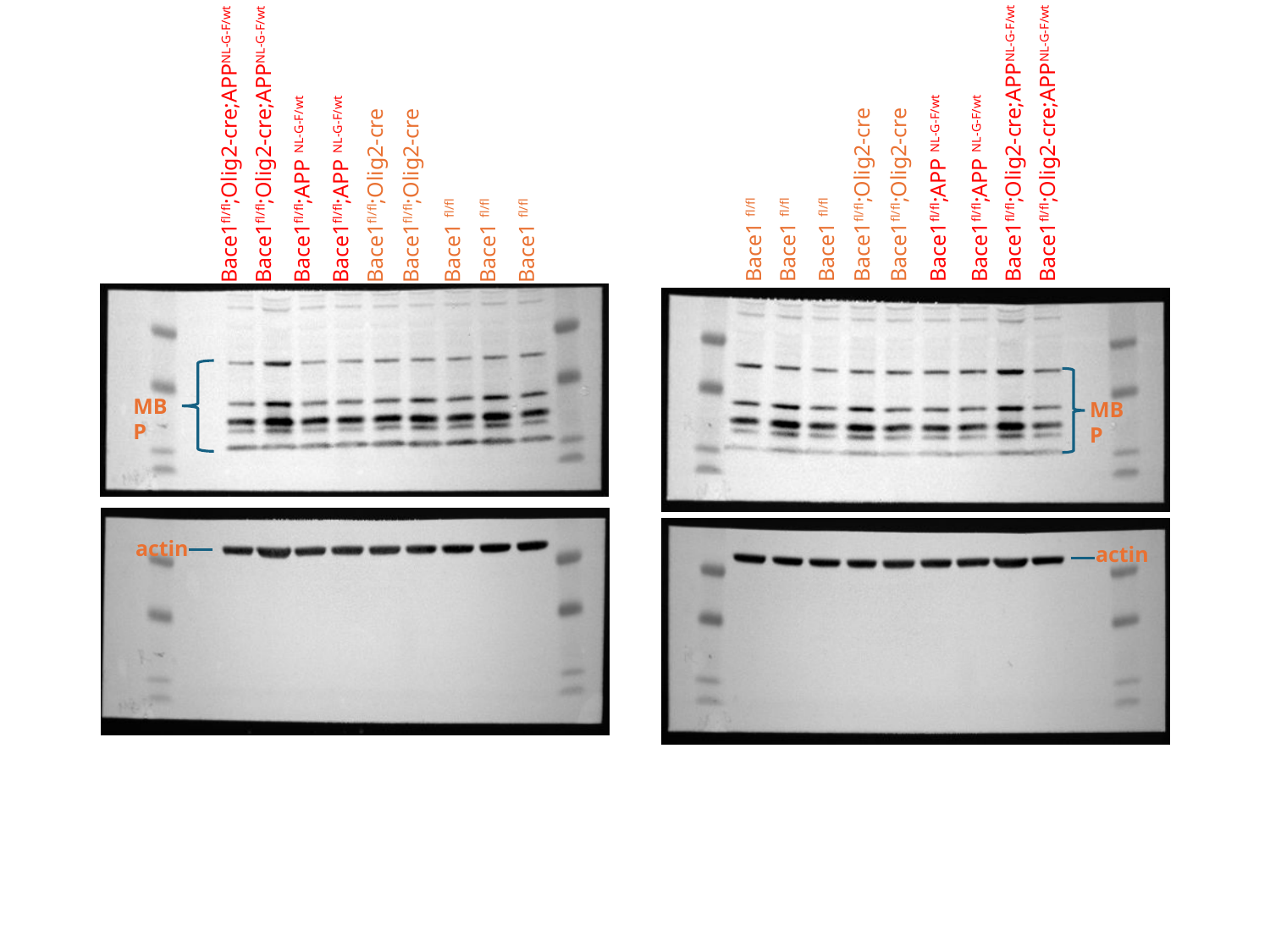

Bace1fl/fl;Olig2-cre;APPNL-G-F/wt
Bace1fl/fl;Olig2-cre;APPNL-G-F/wt
Bace1fl/fl;Olig2-cre;APPNL-G-F/wt
Bace1fl/fl;Olig2-cre;APPNL-G-F/wt
Bace1fl/fl;Olig2-cre
Bace1fl/fl;Olig2-cre
Bace1fl/fl;Olig2-cre
Bace1fl/fl;Olig2-cre
Bace1fl/fl;APP NL-G-F/wt
Bace1fl/fl;APP NL-G-F/wt
Bace1fl/fl;APP NL-G-F/wt
Bace1fl/fl;APP NL-G-F/wt
Bace1 fl/fl
Bace1 fl/fl
Bace1 fl/fl
Bace1 fl/fl
Bace1 fl/fl
Bace1 fl/fl
MBP
MBP
actin
actin

## Slide 4
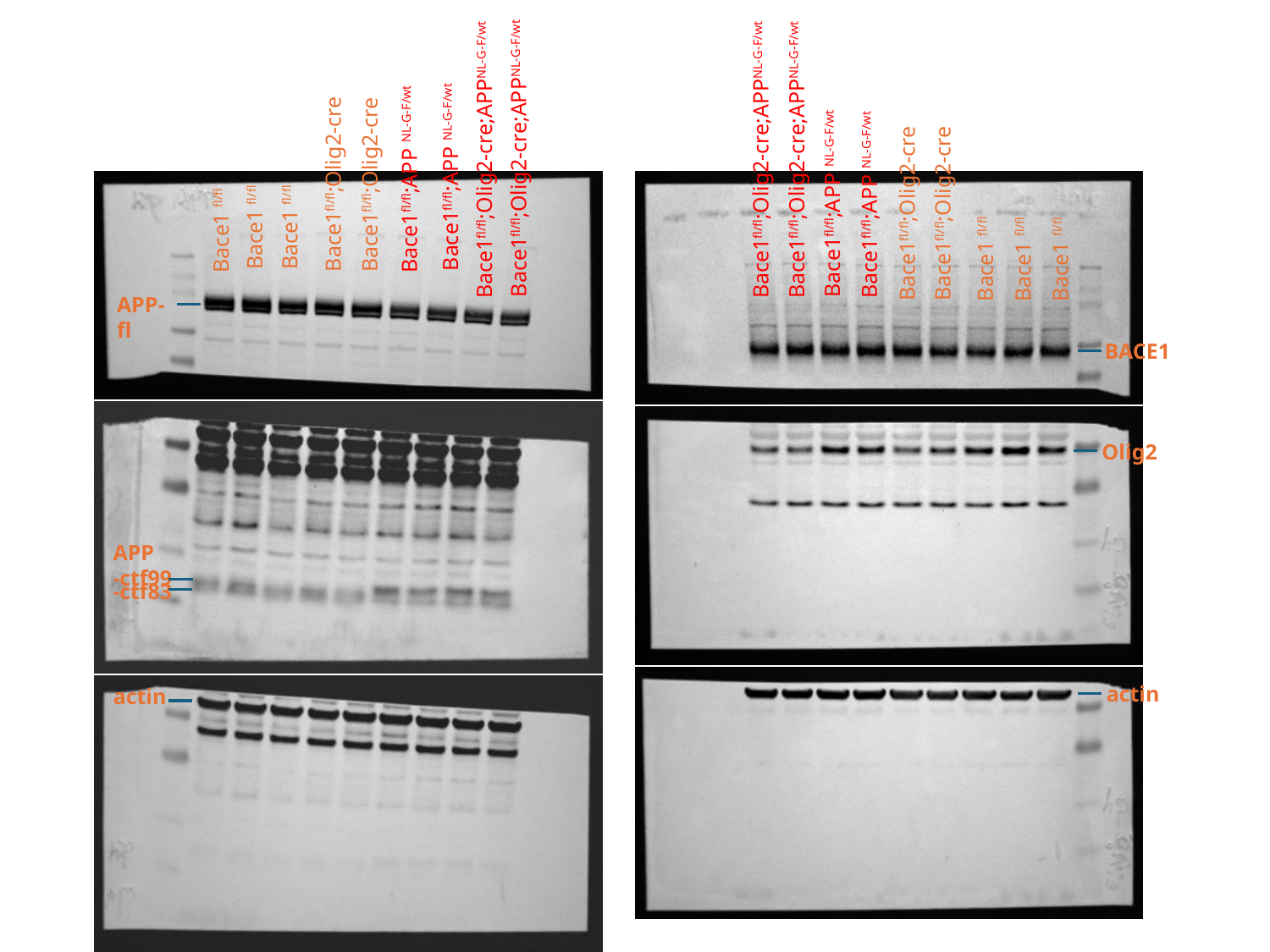

Bace1fl/fl;Olig2-cre;APPNL-G-F/wt
Bace1fl/fl;Olig2-cre;APPNL-G-F/wt
Bace1fl/fl;Olig2-cre
Bace1fl/fl;Olig2-cre
Bace1fl/fl;APP NL-G-F/wt
Bace1fl/fl;APP NL-G-F/wt
Bace1 fl/fl
Bace1 fl/fl
Bace1 fl/fl
BACE1
Olig2
actin
Bace1fl/fl;Olig2-cre;APPNL-G-F/wt
Bace1fl/fl;Olig2-cre;APPNL-G-F/wt
Bace1fl/fl;Olig2-cre
Bace1fl/fl;Olig2-cre
Bace1fl/fl;APP NL-G-F/wt
Bace1fl/fl;APP NL-G-F/wt
Bace1 fl/fl
Bace1 fl/fl
Bace1 fl/fl
APP-fl
APP
-ctf99
-ctf83
actin

## Slide 5
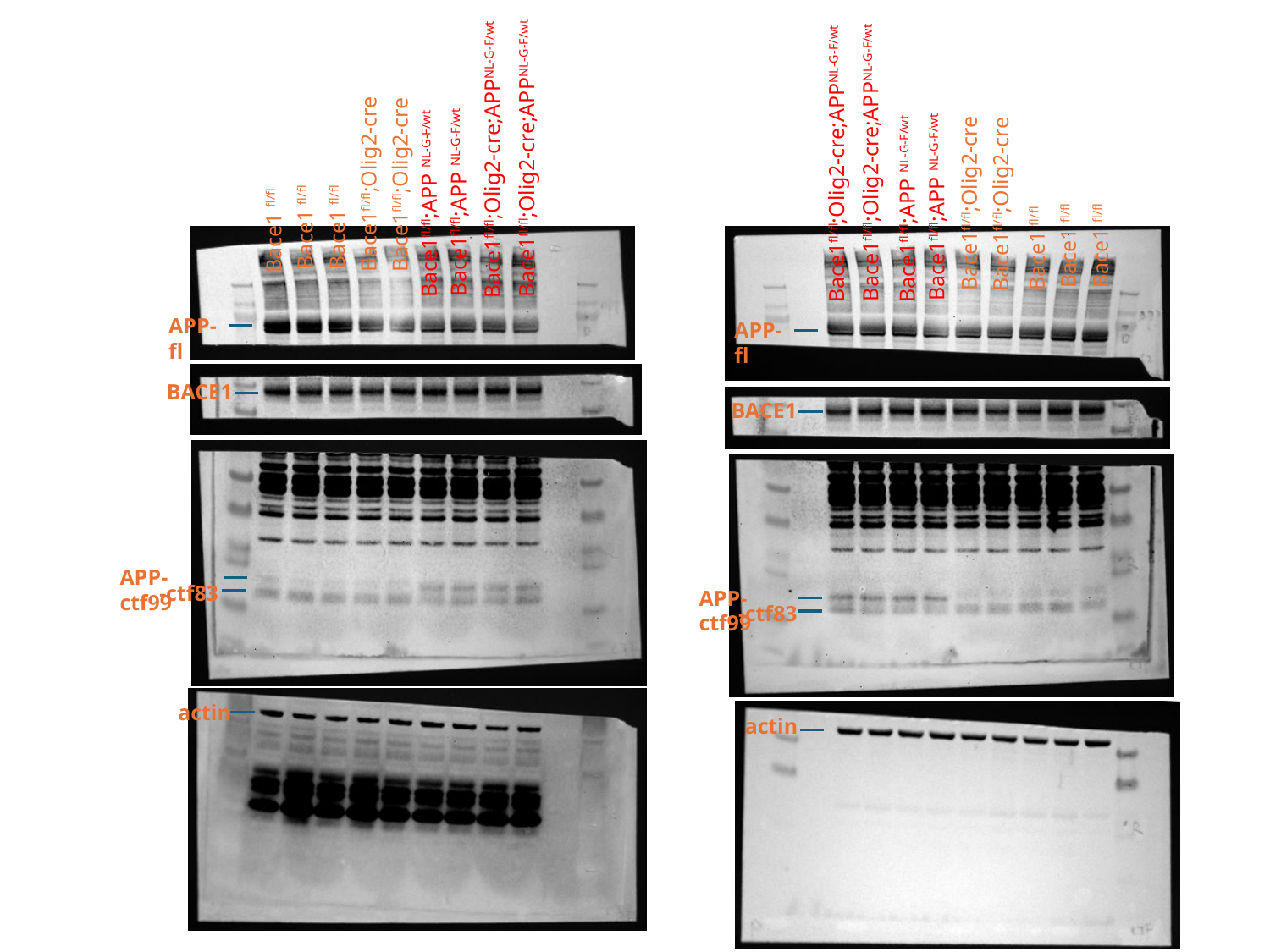

Bace1fl/fl;Olig2-cre;APPNL-G-F/wt
Bace1fl/fl;Olig2-cre;APPNL-G-F/wt
Bace1fl/fl;Olig2-cre
Bace1fl/fl;Olig2-cre
Bace1fl/fl;APP NL-G-F/wt
Bace1fl/fl;APP NL-G-F/wt
Bace1 fl/fl
Bace1 fl/fl
Bace1 fl/fl
APP-fl
BACE1
APP-ctf99
-ctf83
actin
Bace1fl/fl;Olig2-cre;APPNL-G-F/wt
Bace1fl/fl;Olig2-cre;APPNL-G-F/wt
Bace1fl/fl;Olig2-cre
Bace1fl/fl;Olig2-cre
Bace1fl/fl;APP NL-G-F/wt
Bace1fl/fl;APP NL-G-F/wt
Bace1 fl/fl
Bace1 fl/fl
Bace1 fl/fl
APP-fl
BACE1
APP-ctf99
-ctf83
actin

## Slide 6
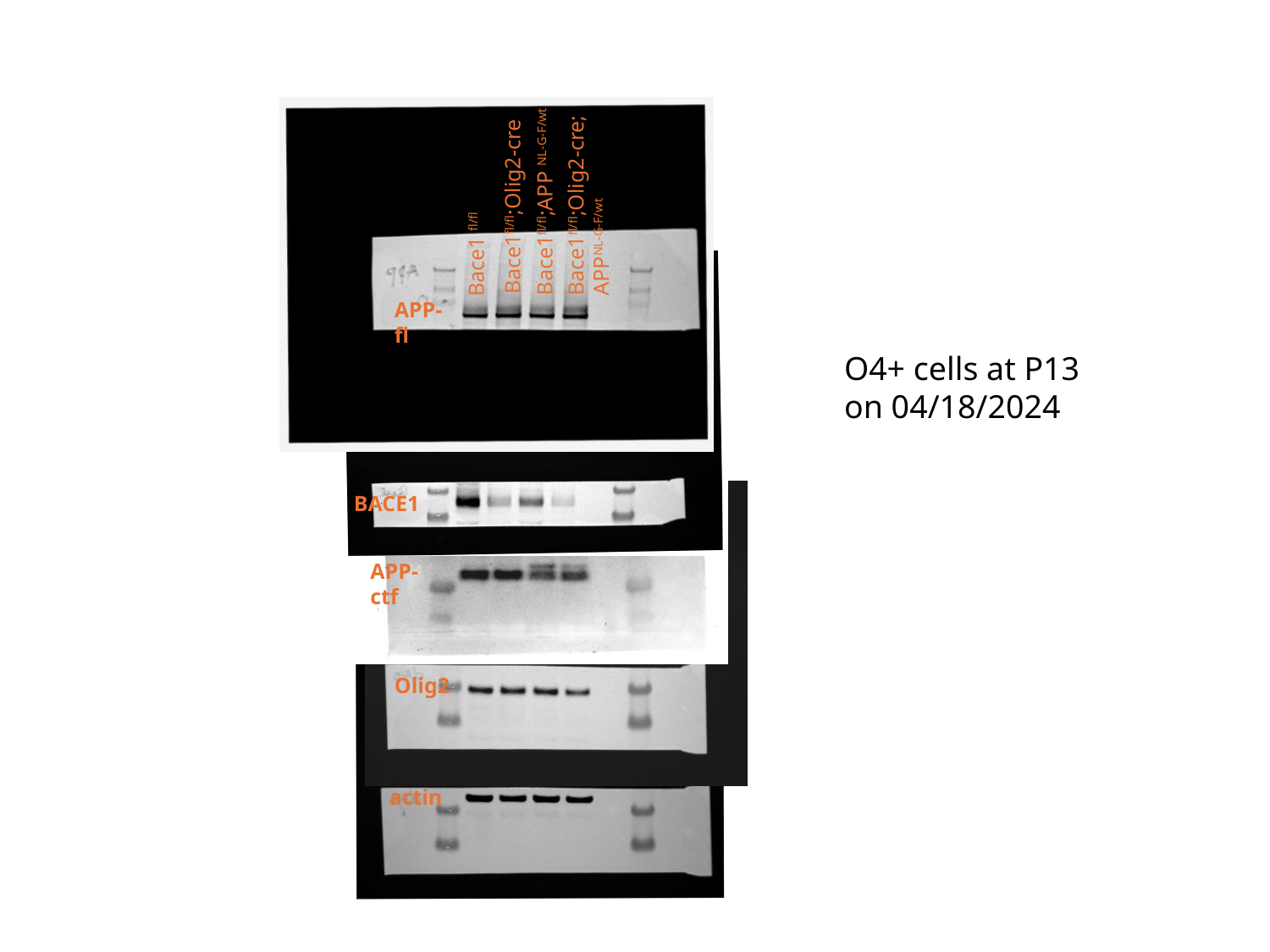

Bace1fl/fl;Olig2-cre
Bace1fl/fl;Olig2-cre;
APPNL-G-F/wt
Bace1fl/fl;APP NL-G-F/wt
Bace1 fl/fl
APP-fl
BACE1
APP-ctf
Olig2
actin
O4+ cells at P13
on 04/18/2024

## Slide 7
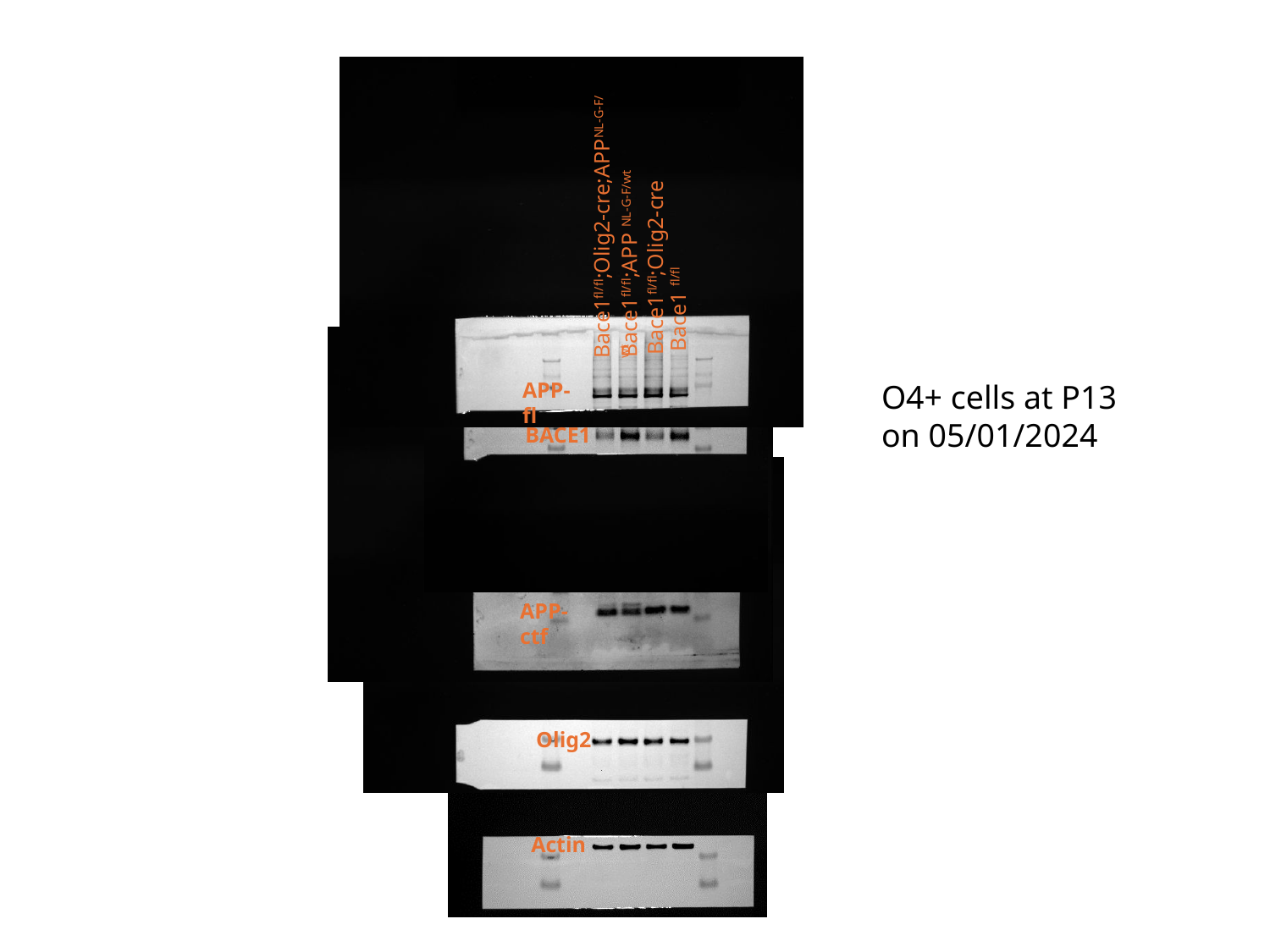

Bace1fl/fl;Olig2-cre;APPNL-G-F/wt
Bace1fl/fl;APP NL-G-F/wt
Bace1fl/fl;Olig2-cre
Bace1 fl/fl
APP-fl
BACE1
APP-ctf
Olig2
Actin
O4+ cells at P13
on 05/01/2024

## Slide 8
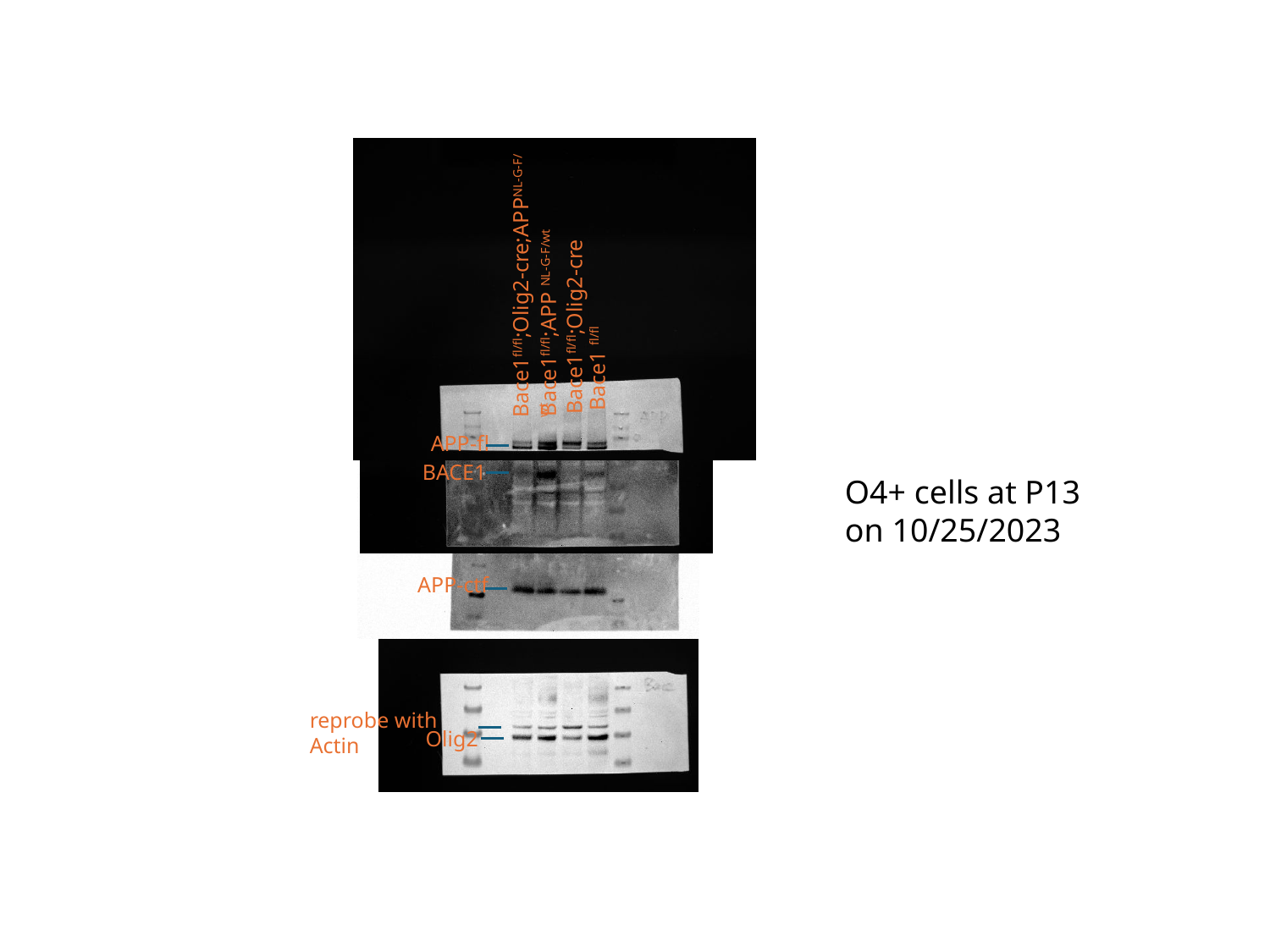

Bace1fl/fl;Olig2-cre;APPNL-G-F/wt
Bace1fl/fl;APP NL-G-F/wt
Bace1fl/fl;Olig2-cre
Bace1 fl/fl
APP-fl
BACE1
APP-ctf
reprobe with Actin
Olig2
O4+ cells at P13
on 10/25/2023

## Slide 9
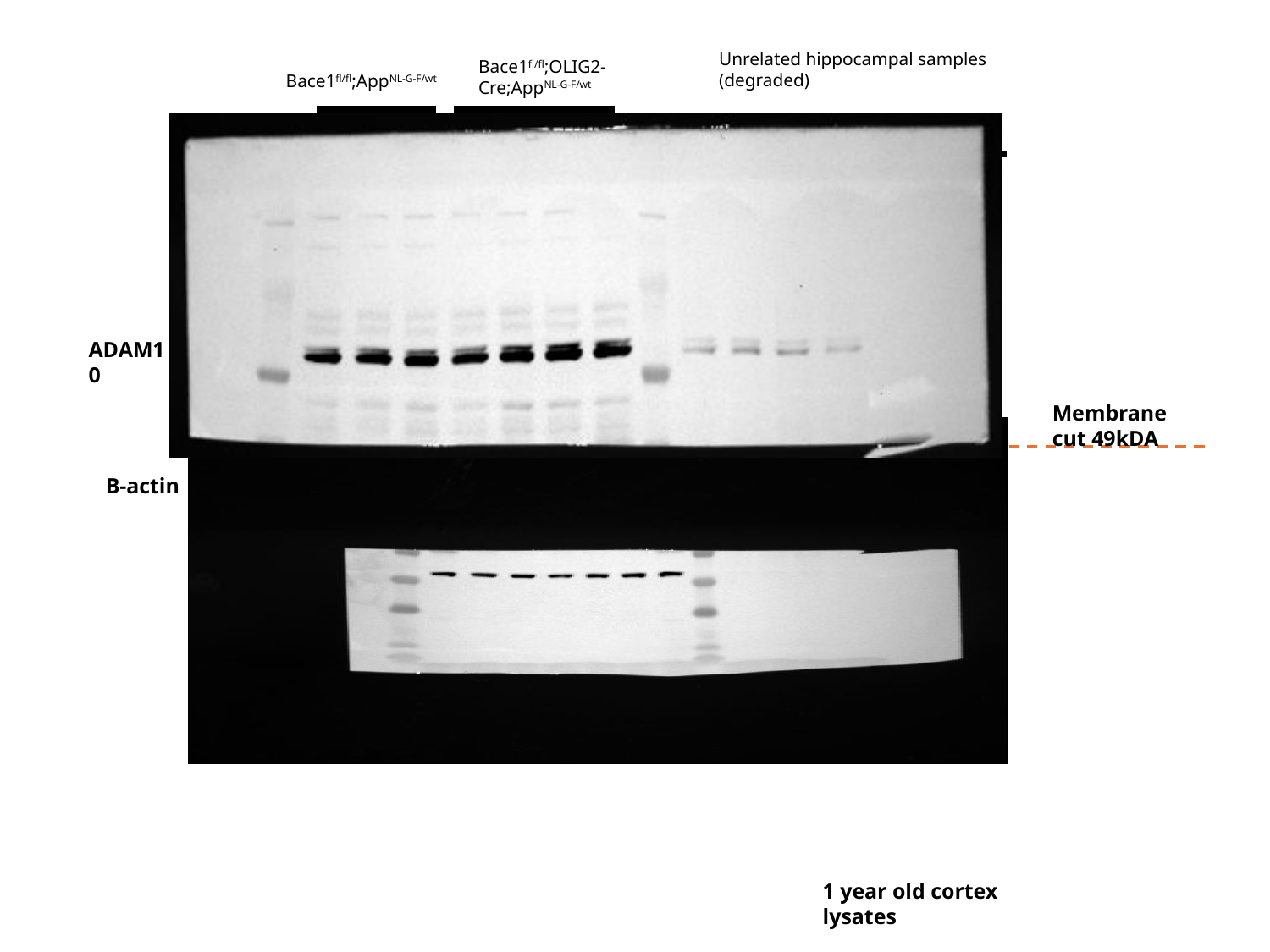

Unrelated hippocampal samples (degraded)
Bace1fl/fl;OLIG2-Cre;AppNL-G-F/wt
Bace1fl/fl;AppNL-G-F/wt
ADAM10
Membrane cut 49kDA
B-actin
1 year old cortex lysates

## Slide 10
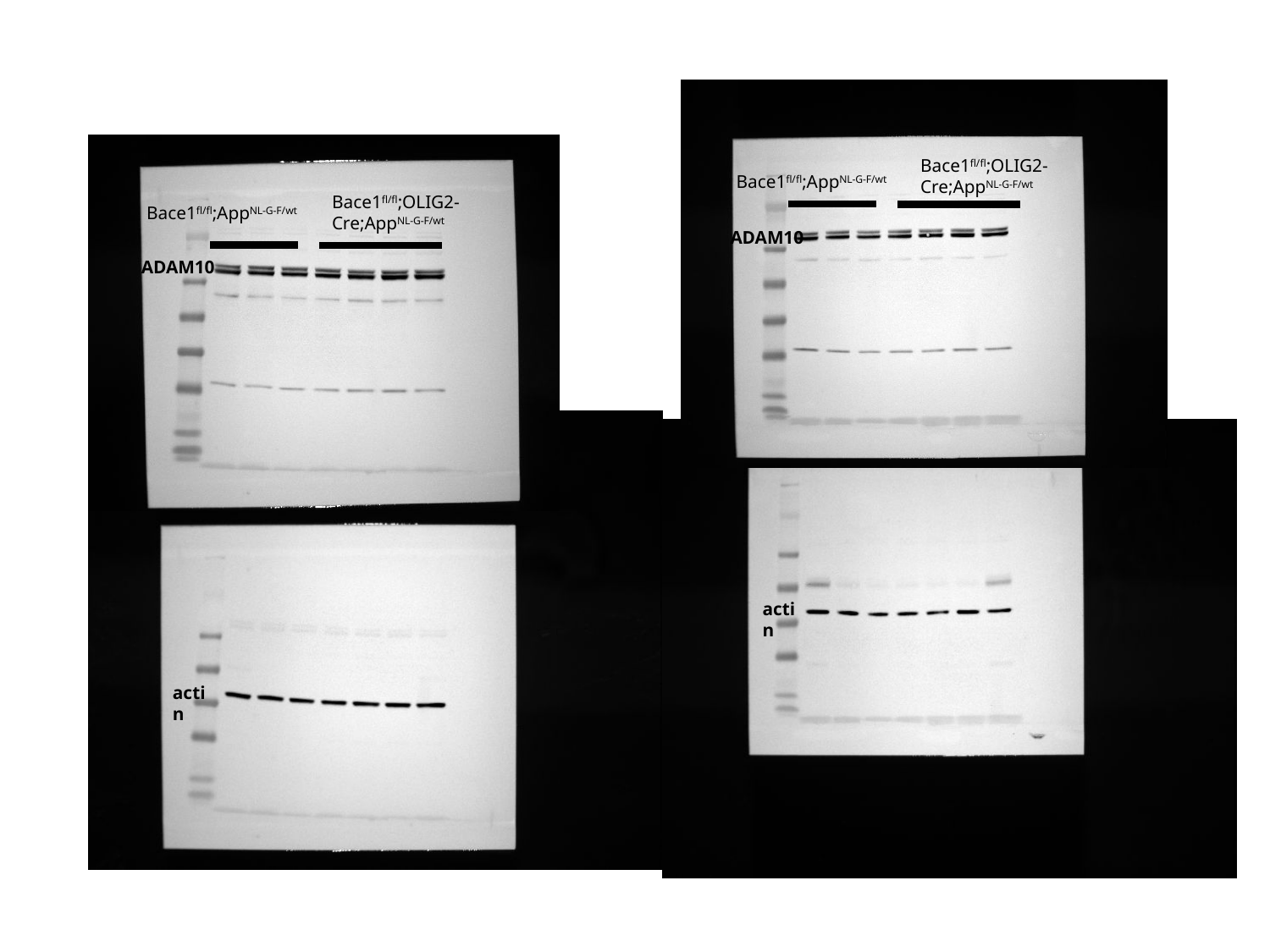

Bace1fl/fl;OLIG2-Cre;AppNL-G-F/wt
Bace1fl/fl;AppNL-G-F/wt
Bace1fl/fl;OLIG2-Cre;AppNL-G-F/wt
Bace1fl/fl;AppNL-G-F/wt
ADAM10
ADAM10
actin
actin
